# The impact of household structure on disease-induced herd immunity

**DOI:** 10.1007/s00285-023-02010-7

**Published:** 2023-11-08

**Authors:** Frank Ball, Liam Critcher, Peter Neal, David Sirl

**Affiliations:** School of Mathematical Sciences, University of Nottingham, University Park, Nottingham, NG7 2RD UK

**Keywords:** Disease-induced herd immunity level, Household epidemic model, SEIR epidemic, Vaccine-induced herd immunity level, 92D30

## Abstract

The disease-induced herd immunity level $h_D$ is the fraction of the population that must be infected by an epidemic to ensure that a new epidemic among the remaining susceptible population is not supercritical. For a homogeneously mixing population $h_D$ equals the classical herd immunity level $h_C$, which is the fraction of the population that must be vaccinated in advance of an epidemic so that the epidemic is not supercritical. For most forms of heterogeneous mixing $h_D<h_C$, sometimes dramatically so. For an SEIR (susceptible $\rightarrow$ exposed $\rightarrow$ infective $\rightarrow$ recovered) model of an epidemic among a population that is partitioned into households, in which individuals mix uniformly within households and, in addition, uniformly at a much lower rate in the population at large, we show that $h_D>h_C$ unless variability in the household size distribution is sufficiently large. Thus, introducing household structure into a model typically has the opposite effect on disease-induced herd immunity than most other forms of population heterogeneity. We reach this conclusion by considering an approximation ${\tilde{h}}_D$ of $h_D$, supported by numerical studies using real-world household size distributions. For $n=2, 3$, we prove that ${\tilde{h}}_D>h_C$ when all households have size *n*, and conjecture that this inequality holds for any common household size *n*. We prove results comparing ${\tilde{h}}_D$ and $h_C$ for epidemics which are highly infectious within households, and also for epidemics which are weakly infectious within households.

## Introduction

During the ongoing COVID-19 pandemic there has been considerable discussion of herd immunity. For a very wide range of epidemic models, specifically models for which the basic reproduction number $R_0$ is given by the maximal eigenvalue of a next-generation matrix, if $R_0$ is greater than one, then vaccinating a fraction $h_C=1-R_0^{-1}$ of the population, chosen uniformly at random, with a perfect vaccine (i.e. one that necessarily renders its recipient immune to the disease) in advance of an outbreak reduces the reproduction number to one and thus prevents a large outbreak (see, for example, Diekmann et al. (2013), page 199). The quantity $h_C$ is the classical (or vaccination-induced) herd immunity level. For a disease in which infection confers immunity to subsequent infection, herd immunity can also be attained by letting an epidemic run its natural course, possibly with some restrictions in place, for example, lockdown or other non-pharmaceutical interventions. The disease-induced herd immunity level $h_D$ is the fraction of the population that needs to be infected before the effective basic reproduction number (i.e. $R_0$ for an epidemic among the remaining susceptible individuals) is reduced to one. For definiteness, we define $h_D$ assuming no restrictions are in place and the epidemic simply runs its natural course.

For an epidemic among a homogeneously mixing population, the classical and disease-induced herd immunity levels are equal. However, that typically is not the case for epidemics among heterogeneous populations. For example, Britton et al. (2020) showed that in a model for COVID-19 in which the population was structured by age and activity level, when $R_0=2.5$, the disease-induced herd immunity level $h_D=0.43$, which is substantially lower than $h_C=0.6$, and Gomes et al. (2022) obtained even lower values for $h_D$ in a model where individuals varied in susceptibility. These observations have a simple intuitive explanation. For example, in the model with varying susceptibility, individuals with higher susceptibility are more likely to be infected early in the epidemic and consequently the average susceptibility of the remaining susceptibles decreases as the epidemic progresses leading to $h_D<h_C$. It seems likely that similar arguments hold for many other forms of heterogeneities, with the general conclusion that introducing heterogeneity into a model has the effect of reducing the disease-induced herd immunity level $h_D$.

An important population structure for epidemics among human populations, which can have a significant impact on disease dynamics and the performance of vaccination strategies, is that induced by households. The aim of this paper is to investigate the impact of household structure on the disease-induced herd immunity level. We use an extension of the SIR (susceptible $\rightarrow$ infective $\rightarrow$ recovered) model introduced by Ball et al. (1997) to include an exposed (latent) period. In this model, infective individuals make two types of infectious contacts: *local* contacts with individuals chosen uniformly at random from their household and, at a much lower rate, *global* contacts with individuals chosen uniformly at random from the whole population. In sharp contrast to most other forms of heterogeneity, we find that the effect of household structure is generally to *increase* $h_D$.

For most models it is difficult to calculate $h_D$ as it requires knowledge of the trajectory of the epidemic, which typically is not available in closed form. Moreover, in a stochastic model the disease-induced herd immunity level is in fact a random variable, as it depends on the realised trajectory, which converges to $h_D$ as the population size converges to infinity. The following approximation to $h_D$, which we adapt to our model, is used in Britton et al. (2020) in the context of a multitype SIR epidemic model. A new model is considered in which *all* transmission rates are multiplied by a factor $\kappa <1$ and its (limiting deterministic) final outcome is determined. (Note that this factor is denoted by $\alpha$ in Britton et al. (2020).) Let ${\hat{\kappa }}$ be the value of $\kappa$ so that the effective $R_0$ among the remaining susceptibles is one. Then the fraction of the population ultimately infected by the epidemic with $\kappa ={\hat{\kappa }}$ gives an approximation to $h_D$, which we denote by ${\tilde{h}}_D$. Note that ${\tilde{h}}_D$ is not affected by the introduction of a latent period into the model, as the distribution of the final size of a stochastic SEIR (susceptible $\rightarrow$ exposed $\rightarrow$ infective $\rightarrow$ recovered) model is invariant to very general assumptions concerning the latent period. We adopt a similar approach to obtain an approximation ${\tilde{h}}_D$ to $h_D$ for the above households model, except that only global transmission rates are multiplied by $\kappa$, with local transmission rates unchanged.

The above definition of $h_D$ assumes that no restrictions are in place. Let ${\hat{h}}_D$ be a generic notation for the disease-induced herd immunity level under restrictions. In practice, ${\hat{h}}_D$ may depend on the precise pattern of restrictions imposed prior to herd immunity being reached (see, for example, Di Lauro et al. (2021)). A commonly-made assumption in modelling restrictions is that at time $t \ge 0$ *all* transmission rates are multiplied by a factor $\kappa (t)$. Under this assumption, Britton et al. (2021) show that ${\hat{h}}_D$ is independent of $\{\kappa (t): t \ge 0\}$ if mixing is separable. Moreover, for the examples in Britton et al. (2020, 2021), numerical studies showed that the precise timings of restrictions had minimal, if any, effect on ${\hat{h}}_D$. The situation is more subtle if restrictions are not applied uniformly, where for some models ${\hat{h}}_D$ can be highly dependent on the pattern of restrictions (Di Lauro et al. 2021). However, numerical studies indicate that is not the case for the present households model, with restrictions affecting only global transmission rates. Note that ${\tilde{h}}_D={\hat{h}}_D$ when such restrictions with factor ${\hat{\kappa }}$ are applied throughout the epidemic. Numerical studies suggest that, under many restrictions, ${\tilde{h}}_D$ is a better approximation than $h_D$ to ${\hat{h}}_D$.

The usual definition of $R_0$ as the maximal eigenvalue of a next-generation matrix, or in non-mathematical terms as the mean number of infectious contacts made by a typical infective in an otherwise susceptible population, does not hold for the present households SEIR model, since even in the early stages of an epidemic there are likely to be repeat local contacts within a household. Pellis et al. (2012) give an alternative definition of $R_0$ via a linear approximation of the early phase of an epidemic in terms of generations of infections, which coincides with the usual definition when it is applicable but can also be extended to models with small mixing groups such as the households SEIR model. Calculation of $R_0$ for the households SEIR model is quite complex. A simpler to calculate reproduction number for the households SEIR model is $R_*$ (see Ball et al. 1997), which is based on the proliferation of infected households (rather than infected individuals). Precise definitions of $R_0$ and $R_*$ and discussion of their properties are given in Sect. 2.2. The reproduction number $R_*$ is useful for determining herd immunity levels, owing to its ease of calculation. However, it is not comparable between different household structures, unlike $R_0$, because it is a households-based rather than an individual-based reproduction number.

Before describing the main results of the paper, we need some more notation. Let $\lambda _L$ denote the individual-to-individual local infection rate and $\lambda _G$ denote the overall rate that an infective makes global contacts. Further, let *H* be a random variable describing the size of a household chosen uniformly at random and ${\tilde{H}}$, the size-biased version of *H*, be a random variable describing the size of the household to which an individual chosen uniformly at random from the population belongs (see Sect. 2.1). Let $\mu _{{\tilde{H}}}=\textrm{E}[{\tilde{H}}]$. For $i=1,2,\dots$, let $\mu _{{\tilde{H}}}^{[i]}$ denote the *i*th factorial moment of ${\tilde{H}}$ and ${\hat{\mu }}^{[i]}_{{\tilde{H}}}=i!\mu _{{\tilde{H}}}\left( \mu _{{\tilde{H}}}-1\right) ^{i-1}$ be the *i*th factorial moment of a geometric distribution with success probability $\mu _{{\tilde{H}}}^{-1}$.

The complexities of the households model render analytical results comparing ${\tilde{h}}_D$ and $h_C$ hard to obtain in general. First, we consider epidemics which are highly locally infectious, in that if one individual in the household becomes infected then the whole household becomes infected. This assumption, which was introduced in Becker and Dietz (1995), is obtained by letting $\lambda _L = \infty$ in our model. Under this assumption, the following are our main results.

- Theorem 4.1. If all households have size $n>1$ and $R_*>1$, then ${\tilde{h}}_D > h_C$.
- Theorem 4.2. Under the conditions of Theorem 4.1, ${\tilde{h}}_D - h_C$ is maximised as a function of $\lambda _G$ when $R_0=2$.
- Theorem 4.3. ${\tilde{h}}_D = h_C$ for all $\lambda _G$ such that $R_*>1$ if and only if ${\tilde{H}}$ follows a geometric distribution, so *H* follows a logarithmic distribution.
- Theorem 4.4. Suppose that the epidemic is only just above threshold (i.e. $R_*$ is only just above 1) and $l^{*}=\inf _{k \ge 2}\{k: \mu _{{\tilde{H}}}^{[k]} \ne {\hat{\mu }}^{[k]}_{{\tilde{H}}}\}< \infty$. Then ${\tilde{h}}_D>h_C$ if $\mu _{{\tilde{H}}}^{[l^{*}]}<{\hat{\mu }}^{[l^{*}]}_{{\tilde{H}}}$ and ${\tilde{h}}_D<h_C$ if $\mu _{{\tilde{H}}}^{[l^{*}]}>{\hat{\mu }}^{[l^{*}]}_{{\tilde{H}}}$.
- Corollary 4.5. Under the conditions of Theorem 4.4, ${\tilde{h}}_D>h_C$ if $\textrm{var}({\tilde{H}})<\textrm{E}[{\tilde{H}}]\textrm{E}[{\tilde{H}}-1]$ and ${\tilde{h}}_D<h_C$ if $\textrm{var}({\tilde{H}})>\textrm{E}[{\tilde{H}}]\textrm{E}[{\tilde{H}}-1]$.
- Theorem 4.6 gives an ordering of ${\tilde{h}}_D$ and $h_C$ for epidemics which are both highly locally and highly globally infectious. The result is not given explicitly here as it requires appreciable further notation.
- Theorem 4.7. Suppose that $n>1$ and $\textrm{P}({\tilde{H}}=n)=p=1-\textrm{P}({\tilde{H}}=1)$, where $0<p<1$, so a fraction *p* of individuals reside in households of size *n* and the remainder in households of size 1. If $n=2$, then ${\tilde{h}}_D>h_C$ for all *p*. For $n\ge 3$, if $p \le \frac{n-2}{2(n-1)}$ then ${\tilde{h}}_D<h_C$ for all $\lambda _G$. If $p>\frac{n-2}{2(n-1)}$ then there exists $\lambda _G^{*}(n,p)$ such that ${\tilde{h}}_D>h_C$ for $\lambda _G<\lambda _G^{*}(n,p)$ and ${\tilde{h}}_D<h_C$ for $\lambda _G>\lambda _G^{*}(n,p)$. An expression for $\lambda _G^{*}(n,p)$ involving the root of an algebraic equation is given in Theorem 4.7. If *p* is close to 1 (i.e. the households are nearly all of size *n*) then $\lambda _G$, and thus $R_0$, must be exceedingly large in order to obtain ${\tilde{h}}_D<h_C$.

Analysis is also possible for epidemics that are weakly locally infectious, i.e. when $\lambda _L$ is close to 0. We assume without loss of generality that the infectious period $T_I$ has mean 1, and write ${\tilde{h}}_D(\lambda _L)$ and $h_C(\lambda _L)$ to show explicitly the dependence of these herd immunity levels on $\lambda _L$. Note that the model reduces to a standard homogeneously mixing SEIR epidemic when $\lambda _L=0$, for which $R_0=\lambda _G$ since $\textrm{E}[T_I]=1$. Thus, ${\tilde{h}}_D(0)=h_C(0)=1-R_0^{-1}=1-\lambda _G^{-1}$. Suppose that $\lambda _G>1$, so $R_*>1$, and let $\pi (0)=\frac{1}{\lambda _G}$.

- Theorem 4.8. As $$\lambda _L \downarrow 0$$,
  $$\begin{aligned}{\tilde{h}}_D(\lambda _L)-h_C(\lambda _L)=2\lambda _L^2\pi (0)^2(1-\pi (0))\left[ \textrm{E}[{\tilde{H}}-1]-\textrm{var}({\tilde{H}})\right] +o(\lambda _L^2).\end{aligned}$$
- Corollary 4.9. If $\textrm{var}({\tilde{H}}) < \textrm{E}[{\tilde{H}}-1]$ then ${\tilde{h}}_D>h_C$ for all sufficiently small $\lambda _L>0$. If $\textrm{var}({\tilde{H}}) > \textrm{E}[{\tilde{H}}-1]$ then ${\tilde{h}}_D<h_C$ for all sufficiently small $\lambda _L>0$.
- Corollary 4.10. Suppose all households are the same size $n>1$. Then, for all sufficiently small $\lambda _L>0$, we have ${\tilde{h}}_D>h_C$.

The final theorem concerns the case when $0< \lambda _L < \infty$ and all households have the same size *n*.

- Theorem 4.11. For a common household size $n=2$ or $n=3$, and for any $\lambda _G$ and $\lambda _L>0$ such that $R_*>1$, we have ${\tilde{h}}_D>h_C$.

We conjecture, supported by numerical studies, that Theorem 4.11 holds for all $n>1$.

The remainder of the paper is structured as follows. In Sect. 2, we define the stochastic SEIR households model underlying our analysis, describe briefly its threshold behaviour, calculation of the reproduction numbers, $R_*$ and $R_0$, and of the final outcome in the event of an epidemic taking off. We also present a deterministic model which approximates epidemics that take off. In Sect. 3, we describe calculation of the vaccine-induced herd immunity level $h_C$, discuss the definition of the disease-induced herd immunity level $h_D$ and describe in detail its approximation ${\tilde{h}}_D$. Theorems concerning comparison of ${\tilde{h}}_D$ and $h_C$ are given in Sect. 4, with some of the longer proofs being deferred to an appendix. Numerical comparisons of herd immunity levels are given in Sect. 5, including illustration of theorems and study of herd immunity levels for real-world household size distributions. In Sect. 6, we give some concluding comments and discuss possible directions for future research.

## SEIR households model

### Model definition

We consider an SEIR (susceptible $\rightarrow$ exposed $\rightarrow$ infective $\rightarrow$ recovered) model for an epidemic among a closed and finite population separated into households. This is similar to the model in Ball et al. (1997) with an extra (exposed) state. The household structure is given as follows. We suppose, for $n=1,2,\dots$, there are $m_n$ households of size *n*. There are $m=\sum _{n=1}^{\infty }m_n$ households (with $m<\infty$), and the total population size is $N=\sum _{n=1}^{\infty }nm_n$.

The epidemic begins at time $t=0$ with one initial infective (chosen uniformly at random from the population) and with all other members of the population susceptible. When a given susceptible is contacted by an infective, they become exposed (latent infective) for a time that is distributed according to a non-negative random variable $T_E$, with an arbitrary but specified distribution which is almost surely finite. When their exposed period ends, an individual becomes infectious for a time distributed according to a non-negative random variable $T_I$, with an arbitrary but specified distribution having finite mean. During their infectious period any given infective makes global contacts with any given susceptible according to a Poisson process with rate $\frac{\lambda _G}{N}$. Further, any given infective makes local contacts with any given susceptibles member of their household according to a Poisson process with rate $\lambda _L$. Once their infectious period ends, an infective recovers and has no further role in the epidemic. When there are no infectives or exposed infectives remaining, the epidemic terminates. Finally, all Poisson processes describing infectious contacts (whether or not either or both of the individuals involved are the same), as well as the random variables for exposed and infectious periods, are assumed to be mutually independent.

Many of the results in the paper are based on approximations which become exact in the limit as the number of households $m \rightarrow \infty$ in an appropriate fashion. For $n=1,2, \dots$, let $\alpha _n^{(m)}=\frac{m_n}{m}$ be the fraction of households that have size *n*. Precise conditions for such asymptotic results are beyond the scope of this paper. We assume that $\lim _{m \rightarrow \infty }\alpha _n^{(m)}=\alpha _n$ $(n=1,2,\dots )$, where $\sum _{n=1}^{\infty } \alpha _n=1$ and $\sum _{n=1}^{\infty } n \alpha _n<\infty$. To ease the presentation we suppress the dependence on *m* of parameters such as $\alpha _n^{(m)}$ and just use their asymptotic values.

### Threshold behaviour

Suppose that the number of households (*m*) is large. Since the epidemic begins with one initial infective, the probability that an individual contacted globally belongs to a previously infected household is small during the early stages of the epidemic. Thus the early stages of the epidemic can be approximated by a branching process, describing the proliferation of infected households, in which every global contact is with a previously uninfected household (Ball 1996). The offspring mean $R_*$ of this branching process, i.e. the expected number of global contacts occurring from a typical contacted household, is a threshold parameter for the households model. Standard branching process theory implies that, in the limit as $m \rightarrow \infty$, the epidemic takes off with strictly positive probability if and only if $R_*>1$. In the event the epidemic takes off, a non-negligible fraction (a large number of households) of the population becomes infected.

The derivation of $R_*$ is as follows. For $n=1,2,\dots$, let ${\tilde{\alpha }}_n=\frac{nm_n}{N}$ be the probability an individual chosen uniformly at random from the population resides in a household of size *n*. Consider a globally contacted individual in an otherwise fully susceptible household of size *n*. This individual begins a local outbreak within their household with dynamics determined by local infection since, in the branching process, global contacts are with previously fully susceptible households. Let $\mu _n(\lambda _L)$ denote the mean size, including the initial infective, of a single-household epidemic with *n* members and local infection rate $\lambda _L$ with only the initial infective infected globally. Global contacts from a given individual occur at rate $\lambda _G$, and such an individual has mean infectious period $\textrm{E}[T_I]$. Wald’s identity for epidemics (see Ball 1986, Theorem 2.1) then gives the mean number of global contacts from a given contacted household of size *n* to be $\mu _n(\lambda _L)\lambda _G\textrm{E}[T_I]$. By conditioning on the size of a household of a contacted individual, we have that

2.1 $$\begin{aligned} R_*=\sum _{n=1}^{\infty }{\tilde{\alpha }}_n\mu _n(\lambda _L)\lambda _G\textrm{E}[T_I]. \end{aligned}$$

In Ball (1986) it is shown that

2.2 $$\begin{aligned} \mu _n(\lambda _L)=n-\sum _{k=1}^{n-1}\left( {\begin{array}{c}n-1\\ k\end{array}}\right) \beta _k(\lambda _L)\phi (k\lambda _L)^{n-k} \;\;\;\;\;(n=1,2,\dots ), \end{aligned}$$

where $\phi (\theta )=\textrm{E}[\textrm{e}^{-\theta T_I}]$ and $\beta _k(\lambda _L)$ $(k=1,2,\dots )$ are defined recursively by

$$\begin{aligned} \sum _{i=1}^{k}\left( {\begin{array}{c}k\\ i\end{array}}\right) \beta _i(\lambda _L)\phi (i\lambda _L)^{k-i}=k\;\;\;\;\;(k=1,2,\dots ). \end{aligned}$$

As explained in Sect. 1, a drawback of $R_*$ is that it is not comparable between models with different household structures. An alternative threshold parameter, which does not suffer from that defect, is the basic reproduction number $R_0$. As also explained in Sect. 1, the usual definition of $R_0$ does not hold for households models. Instead, $R_0$ can be defined by considering generations of infectives, via a directed graph associated with an epidemic (Pellis et al. 2012 and Ball et al. 2016). Such a graph is constructed by having population members as the vertices. If, during their infectious period, individual *x* would contact $x^{\prime }$, a directed edge is drawn from *x* to $x^{\prime }$. The initial infective is the only member of generation 0. The generation of a given individual *x* is the shortest path length from the initial infective to *x*. Note that this may not coincide with real-time generations of infectives. Then $R_0$ is defined by the limit, as the population size goes to infinity, of the asymptotic geometric growth rate of the mean generation size (for a full definition, see Ball et al. (2016), Section 1). For $k=1,2, \dots$ and $i=0,1,\dots ,k-1$, let $\mu _i^{(k)}$ be the mean size of the $i^{\text {th}}$ generation for an epidemic in a household of size *k* with 1 initial infective. (For the present SEIR model, these quantities can be computed using methods described in appendix A of Pellis et al. (2012).) Then $R_0$ in the households model is the unique positive solution $\lambda$ of

2.3 $$\begin{aligned} 1-\lambda _G\textrm{E}[T_I]\sum _{i=0}^{\infty }\frac{\mu _i}{\lambda ^{i+1}}=0, \end{aligned}$$

where $\mu _i=\sum _{n=1}^{\infty }{\tilde{\alpha }}_n\mu _{i}^{(n)}$; see Ball et al. (2016), Section 2.2 for details.

Note that, like $R_*$, the critical value of $R_0$ is 1. More precisely, $R_0=1$ if and only if $R_*=1$; $R_0>1$ if and only if $R_*>1$; and $R_0<1$ if and only if $R_*<1$. We use $R_*$ for calculating or proving results pertaining to herd immunity levels, as it is far simpler to determine than $R_0$. We use $R_0$ when making comparisons between models, owing to its improved interpretability.

### Final outcome

Consider an epidemic initiated by one initial infective in a population of size *N* (*m* households). Let $Z_m$ denote the proportion of individuals infected in the epidemic. Provided $R_*>1$, as $m \rightarrow \infty$, $Z_m$ converges in probability to a discrete random variable *Z* with probability mass function

$$\begin{aligned} \textrm{P}(Z=z) = 1- \textrm{P}(Z=0), \end{aligned}$$

for some $0< z<1$ defined below. (The mass at zero in the random variable *Z* corresponds to the branching process in the previous section going extinct.) We define a major outbreak to have occurred if $Z_m \approx z$ and it follows that the sum of the infectious periods of all infected individuals in a major outbreak, $S_m$, is approximately $N z E[T_I]$. Hence, the probability that a randomly chosen individual avoids global infection during the course of a major outbreak is approximately $\pi = \exp (-\lambda _G E[T_I] z)$, since to avoid global infection there must be no points in a Poisson process of intensity $\lambda _G /N$ run for time $S_m \approx N z E[T_I]$.

Let ${\tilde{\mu }}_n (\lambda _L,\pi )$ denote the mean size of a single-household epidemic in a household of size *n* with local infection rate $\lambda _L$ and $\textrm{Bin} (n,1-\pi )$ initial (globally infected) infectives; using the standard $\textrm{Bin} (n,p)$ notation to denote the binomial distribution. Denote this epidemic model, which is considered in Addy et al. (1991), by ${\tilde{E}}_n(\lambda _L,\pi )$. Returning to the households model, suppose that a major outbreak occurs and let ${\tilde{T}}_n$ denote the total number of individuals infected in a typical household of size *n*, all of whom are initially susceptible. In the limit as $m \rightarrow \infty$, individuals independently avoid global infection with probability $\pi$. Thus in a household of size *n*, $\textrm{Bin} (n,1-\pi )$ will be infected globally and hence $\textrm{E}[{\tilde{T}}_n] = {\tilde{\mu }}_n (\lambda _L, \pi )$. Then equation (3.10) of Ball et al. (1997) yields

$$\begin{aligned} {\tilde{\mu }}_n (\lambda _L, \pi ) = n - \sum _{k=1}^n \left( {\begin{array}{c}n\\ k\end{array}}\right) \phi (k \lambda _L)^{n-k} \pi ^k \beta _k (\lambda _L). \end{aligned}$$

The probability that a given individual in an initially susceptible household of size *n* is infected during the epidemic is approximately ${\tilde{\mu }}_n (\lambda _L, \pi )/n$. Conditioning on the household size of a randomly chosen individual then establishes

2.4 $$\begin{aligned} z = \sum _{n=1}^\infty {\tilde{\alpha }}_n \frac{{\tilde{\mu }}_n (\lambda _L, \pi )}{n}. \end{aligned}$$

Note that for large *m* the effect of the atypical behaviour of the household containing the initial infective becomes negligible and disappears in the limit as $m \rightarrow \infty$. Thus, since $\pi =\exp (-\lambda _G \textrm{E}[T_I] z)$, (2.4) admits an implicit equation for *z*, whereby $z=0$ is always a solution and a second solution $z\in (0,1)$ exists if and only if $R_*>1$. A similar argument can be used to establish, in the event of a major outbreak, the proportions $(P_{n,v})$ $(n=1,2,\ldots ; v=0,1,\ldots ,n)$ of households of size *n* with *v* members ultimately infected. For $n=1,2,\ldots$, $(P_{n,v})$ satisfies the system of equations (see Addy et al. (1991), equation 4)

2.5 $$\begin{aligned} \sum _{i=0}^v \left( {\begin{array}{c}n-i\\ v-i\end{array}}\right) \frac{P_{n,i}}{\phi ((n-v)\lambda _L)^i \pi ^{n-v}} = \left( {\begin{array}{c}n\\ v\end{array}}\right) \qquad (v=0,1,\dots ,n). \end{aligned}$$

The above arguments are made rigorous in Ball et al. (1997), Section 4.2 and hold with or without the inclusion of a latent period, see Ball et al. (1997), Section 3.1.

### Deterministic model

In House and Keeling (2008), Section 2, a system of ordinary differential equations (ODEs) is derived for the evolution over time of the SIR epidemic model with households, assuming that $T_I \sim \textrm{Exp}(\gamma )$, i.e. the infectious period distribution is exponential with mean $\gamma ^{-1}$. House and Keeling’s ODEs represent the deterministic limit of the stochastic process defined in Sect. 2.1 as $m \rightarrow \infty$, under the assumptions that all households are the same size and there is no latent period. We extend the system of ODEs to allow for variable household size and a latent period $T_E\sim \textrm{Exp}(\delta )$.

Consider the model in Sect. 2.1 with maximum household size $n_{\max }$. Let

2.6 $$\begin{aligned} {\mathscr {H}}^{(n_{\max })}=\{(s,e,i,r) \in {\mathbb {Z}}_+^4: 1 \le s+e+i+r\le n_{\max }\}, \end{aligned}$$

where ${\mathbb {Z}}_+=\{0,1,\dots \}$, and for $t\ge 0$ and $(s,e,i,r)\in {\mathscr {H}}^{(n_{\max })}$, denote by $H^{(m)}_{s,e,i,r}(t)$ the number of households with *s* susceptible, *e* exposed, *i* infectious and *r* recovered members at time *t*. For $n=1,2,\dots , n_{\max }$, let

$$\begin{aligned} {\mathscr {H}}_n=\{(s,e,i,r) \in {\mathscr {H}}^{(n_{\max })}: s+e+i+r=n\}. \end{aligned}$$

Then, $\sum _{(s,e,i,r) \in {\mathscr {H}}_n} H^{(m)}_{s,e,i,r}(t) = m_n$ for all $t \ge 0$. Let

2.7 $$\begin{aligned} {\mathscr {H}}^{(n_{\max })}_+=\{(s,e,i,r) \in {\mathscr {H}}^{(n_{\max })}: e+i > 0\} \end{aligned}$$

be the set of states in which there is at least one infective or exposed individual. For $(s,e,i,r) \in {\mathscr {H}}^{(n_{\max })}$, we assume that $\frac{1}{m}H^{(m)}_{s,e,i,r}(0) \rightarrow h_{s,e,i,r}(0)$ as $m \rightarrow \infty$, where $\sum _{(s,e,i,r) \in {\mathscr {H}}^{(n_{\max })}_+}h_{s,e,i,r}(0)>0$, so a strictly positive fraction of the population is initially either exposed or infective in the limit as $m \rightarrow \infty$. Then, under the Markovian assumption, we have that $\frac{1}{m}H^{(m)}_{s,e,i,r}(t)$ converges in probability to a deterministic process $h_{s,e,i,r}(t)$ as $m \rightarrow \infty$; see Ethier and Kurtz (1986), Theorem 11.2.1. Clearly, for $n=1,2,\dots , n_{\max }$, we have $\sum _{(s,e,i,r) \in {\mathscr {H}}_n }h_{s,e,i,r}(t)=\alpha _n$ for all *t*. Let ${\bar{i}}(t)=\sum _{(s,e,i,r) \in {\mathscr {H}}^{(n_{\max })}}ih_{s,e,i,r}(t)$. The deterministic model can be obtained by considering the possible transition rates between household states and yields, for $(s,e,i,r) \in {\mathscr {H}}^{(n_{\max })},$

2.8 $$\begin{aligned} \begin{aligned} \frac{d}{dt}{h}_{s,e,i,r} = \;&\delta \left( -eh_{s,e,i,r} +(e+1)h_{s,e+1,i-1,r}\right) \\&+ \gamma \left( -ih_{s,e,i,r}+(i+1)h_{s,e,i+1,r-1} \right) \\&+ \lambda _G{\bar{i}}(t)\left( -sh_{s,e,i,r}+(s+1)h_{s+1,e-1,i,r} \right) \\&+ \lambda _L\left( -sih_{s,e,i,r}+(s+1)ih_{s+1,e-1,i,r} \right) , \end{aligned} \end{aligned}$$

where, on the right-hand side, $h_{s',e',i',r'}(t)=0$ if $(s',e',i',r') \notin {\mathscr {H}}^{(n_{\max })}$.

## Herd immunity in SEIR households model

In this section we outline the various versions of herd immunity that we consider. We start by recapping vaccine-induced herd immunity; we then describe and explore the details of the disease-induced herd immunity level $h_D$ and its approximation ${\tilde{h}}_D$ which are outlined in Sect. 1. In this section, and throughout the remainder of the manuscript, we assume that $R_*>1$, since if this is not the case then herd immunity is already achieved.

### Vaccine-induced herd immunity level $h_C$

Suppose some members of the population are vaccinated before an epidemic occurs. Assume that such a vaccine is given to each member of the population independently with probability *c*, and is perfect, so that vaccinated individuals are completely immune to infection. Then, for $v=0,1,\dots , n$, a given household of size *n* has *v* members vaccinated according to the (binomial) probability $$\left( {\begin{array}{c}n\\ v\end{array}}\right) c^{v}(1-c)^{n-v}$$. We obtain a post-vaccination threshold parameter ${\hat{R}}_U(c)$ by considering a branching process of potential global contacts. If a potential global contact is with a susceptible individual then it triggers a local epidemic; if the contact is with a vaccinated individual then nothing happens.

The mean number of potential global contacts emanating from a single-household epidemic for a household in state (*n*, *v*) that is contacted globally, with the initial infective chosen uniformly at random from members of the household ($\mu _{n,v}$, say) is given by

$$\begin{aligned} \mu _{n,v}=\left( \frac{n-v}{n}\right) \mu _{n-v}(\lambda _L)\lambda _G\textrm{E}[T_I], \end{aligned}$$

for $n=1,2,\dots$ and $v=0,1,\dots , n$. This is because a vaccinated member being contacted leads to no global contacts at all, and an unvaccinated member being contacted initiates a single-household epidemic amongst the $n-v$ non-immune members. Such an unvaccinated individual is contacted with probability $\frac{n-v}{n}$. Conditioning on the vaccination status of an individual’s household, as well as the individual’s household size, and using the same argument as for equation (2.1) yields a post-vaccination threshold parameter

3.1 $$\begin{aligned} {\hat{R}}_U(c)=\sum _{n=1}^{\infty }{\tilde{\alpha }}_n\sum _{v=0}^{n}\left( {\begin{array}{c}n\\ v\end{array}}\right) c^{v} (1-c)^{n-v}\left( \frac{n-v}{n}\right) \mu _{n-v}(\lambda _L)\lambda _G\textrm{E}[T_I], \end{aligned}$$

with $\mu _0(\lambda _L)=0$. The function ${\hat{R}}_U(c)$ is continuous and strictly decreasing, with ${\hat{R}}_U(0)=R_*>1$ and ${\hat{R}}_U(1)=0$. (To show that ${\hat{R}}_U(c)$ is strictly decreasing, let $$f_n(c)=\sum _{v=0}^{n}\left( {\begin{array}{c}n\\ v\end{array}}\right) c^{v}(1-c)^{n-v}(n-v)\mu _{n-v}(\lambda _L)$$. Then $f_n(c)=\textrm{E}[g(X)]$, where $g(x)=x\mu _x(\lambda _L)$ and $X \sim \textrm{Bin} (n,1-c)$. The function *g* is strictly increasing and *X* is stochastically decreasing in *c*. Thus, $f_n(c)$ is strictly decreasing in *c*, whence so is ${\hat{R}}_U(c)$.) This implies there is a critical value, $h_C$ say, such that ${\hat{R}}_U(h_C)=1$ and a major outbreak can be avoided. The quantity $h_C$ is the vaccine-induced herd immunity level. Note that by ensuring ${\hat{R}}_U(c)\le 1$, the whole population is considered protected as a major outbreak is no longer possible. This argument regarding uniform vaccination is considered (among other vaccination strategies) in Ball and Lyne (2006).

As noted at the start of Sect. 1, for epidemic models in which $R_0$ is given by the maximal eigenvalue of a next generation matrix, $h_C=1-R_0^{-1}$. For the present households model, $R_0$ is computed differently, see Sect. 2.2; if $\lambda _L \in (0, \infty )$ then it follows from Ball et al. (2016), Theorem 1, that $h_C \ge 1-R_0^{-1}$ with equality if and only if $n_{\max } \le 3$. In the highly locally infectious case ($\lambda _L = \infty$), $h_C=1-R_0^{-1}$ for all $n_{\max }$; see Remark 2 following the aforementioned Theorem 1. If $\lambda _L=0$, the model reduces to a standard homogeneously mixing SEIR epidemic and $h_C=1-R_0^{-1}$.

### Disease-induced herd immunity

#### Limiting disease-induced herd immunity level $h_D$

An alternative method of achieving herd immunity in a population arises from the spread of a first wave of infection, in which infected members from the first wave are considered immunised thereafter.

Consider the SIR version of the households model described in Sect. 2.1, with $T_I\sim \textrm{Exp}(\gamma )$ and assume this epidemic takes off. As the epidemic progresses some members of the population are infected, lowering the overall susceptibility of the population. Suppose that the first epidemic is stopped (i.e. all infectious spread, including that within households, is stopped) at time $t>0$. Consider a second epidemic initiated at time *t* with one initial infective and all those individuals infected by time *t* in the first epidemic immune to infection in the second. Recalling that *m* is the number of households in the population, let $R^{(m)}_V(t)$ be the threshold parameter ($R_*$) for this second epidemic, which is a random variable owing to its dependence on the trajectory of the first epidemic. If $R_V^{(m)}(t)\le 1$, then the second epidemic is not supercritical and a major outbreak cannot occur. Disease-induced herd immunity is achieved when the trajectory of $R_V^{(m)}(t)$ crosses one. Write $T^{(m)}_{*}=\inf \{t\ge 0: R_V^{(m)}(t)\le 1\}$ and denote the fraction of the population that is still susceptible at time *t* in the first epidemic by $S^{(m)}(t)$. Then the disease-induced herd immunity level $H_D^{(m)}$ is given by $H_D^{(m)}=1-S^{(m)}\left( T_*^{(m)}\right)$ and is also a random variable determined by the trajectory of the first epidemic. We conjecture that $H_D^{(m)}{\mathop {\longrightarrow }\limits ^{\textrm{p}}}h_D$ as $m\rightarrow \infty$, where $h_D$ is a constant, and in the presence of a latent period $T_E \sim \textrm{Exp}(\delta )$, that $H_D^{(m)}{\mathop {\longrightarrow }\limits ^{\textrm{p}}}h_D^L$ as $m\rightarrow \infty$. Moreover, we conjecture that, under suitable conditions, these convergence results hold also when $T_I$ and $T_E$ follow non-exponential distributions.

To compute $h_D^L$ when $T_I\sim \textrm{Exp}(\gamma )$ and $T_E \sim \textrm{Exp}(\delta )$, we use the deterministic model in Sect. 2.4. Recall the definitions of ${\mathscr {H}}^{(n_{\max })}$ and ${\mathscr {H}}^{(n_{\max })}_+$ at (2.6) and (2.7). Let $\varvec{h}(t)=\left( h_{s,e,i,r}(t): (s,e,i,r) \in {\mathscr {H}}^{(n_{\max })}\right)$ and suppose that $\varvec{h}(0)=\varvec{\epsilon }$. Then the deterministic model given by (2.8) can be used in the obvious fashion to define a disease-induced herd immunity level $h_D^L(\varvec{\epsilon })$. Let ${\mathscr {H}}_{0\text {rec}}=\{(s,e,i,r) \in {\mathscr {H}}^{(n_{\max })}:r=0\}$. We conjecture that $h_D^L(\varvec{\epsilon }^{(k)})\rightarrow h_D^L$ as $k \rightarrow \infty$ for any sequence $(\varvec{\epsilon }^{(k)})$ satisfying $\sum _{(s,e,i,r) \in {\mathscr {H}}^{(n_{\max })}_+}\epsilon _{s,e,i,r}^{(k)} \downarrow 0$ and $\sum _{(s,e,i,r) \in {\mathscr {H}}_{0\text {rec}}} \epsilon _{s,e,i,r}^{(k)} \uparrow 1$ as $k \rightarrow \infty$. Other than in Appendix A, when computing $h_D^L$, we assume that initially a fraction $\epsilon =10^{-5}$ of households are in state $(n_{\max }-1, 0, 1, 0)$, with all other households being fully susceptible. An equivalent assumption is made when computing $h_D$. Note that, if the largest household is of size *n*, the system in (2.8) contains exact order $n^4/24$ equations (or $n^3/6$ in the SIR case) and becomes computationally expensive to solve when *n* is large.

The proofs of the above conjectures require extending the theory of Barbour and Reinert (2013) to the households model and are beyond the scope of this paper. Some brief comments, together with numerical evidence in support of these conjectures are given in Appendix A.

#### Approximate disease-induced herd immunity level ${\tilde{h}}_D$

Calculation of $h_D$ requires deterministic limiting equations, which are not tractable in general; in the Markovian setting, for example, $h_D$ can be found numerically. For other infectious period distributions, such a calculation is generally not available; we consider an approximation (${\tilde{h}}_D$) to $h_D$. We adapt the approach taken in Britton et al. (2020) to the households setting, using the final outcome of an epidemic with reduced global infection rate to approximate the state of the population at the time herd immunity is achieved. The method of approximation is as follows: Let $\kappa$ $\in$ $\left( R_*^{-1},1\right)$. Run to its conclusion an epidemic in which the global infection parameter $\lambda _G$ is replaced by $\kappa \lambda _G$ and all other parameters are unchanged, i.e. an epidemic with prevention measure applied to global infection only. Then expose the population to a second epidemic with $\kappa =1$, with members infected in the first epidemic now immune. The threshold parameter $R_{*}$ for this second epidemic is a function of $\kappa$, which we denote by ${\hat{R}}_{DI}(\kappa )$. Determine ${\hat{\kappa }}$, the smallest value of $\kappa$ such that ${\hat{R}}_{DI}(\kappa )\le 1$ by solving ${\hat{R}}_{DI}({\hat{\kappa }})=1$. Then ${\tilde{h}}_D$ is the fraction of the population infected in the first epidemic with $\kappa ={\hat{\kappa }}$. In summary, we adjust the global infection rate in the first epidemic to force criticality in the second, and then consider the final size of the first epidemic. Note that this method relies on final size results, which are more amenable to study than time-dependent results.

**Fig. 1:**
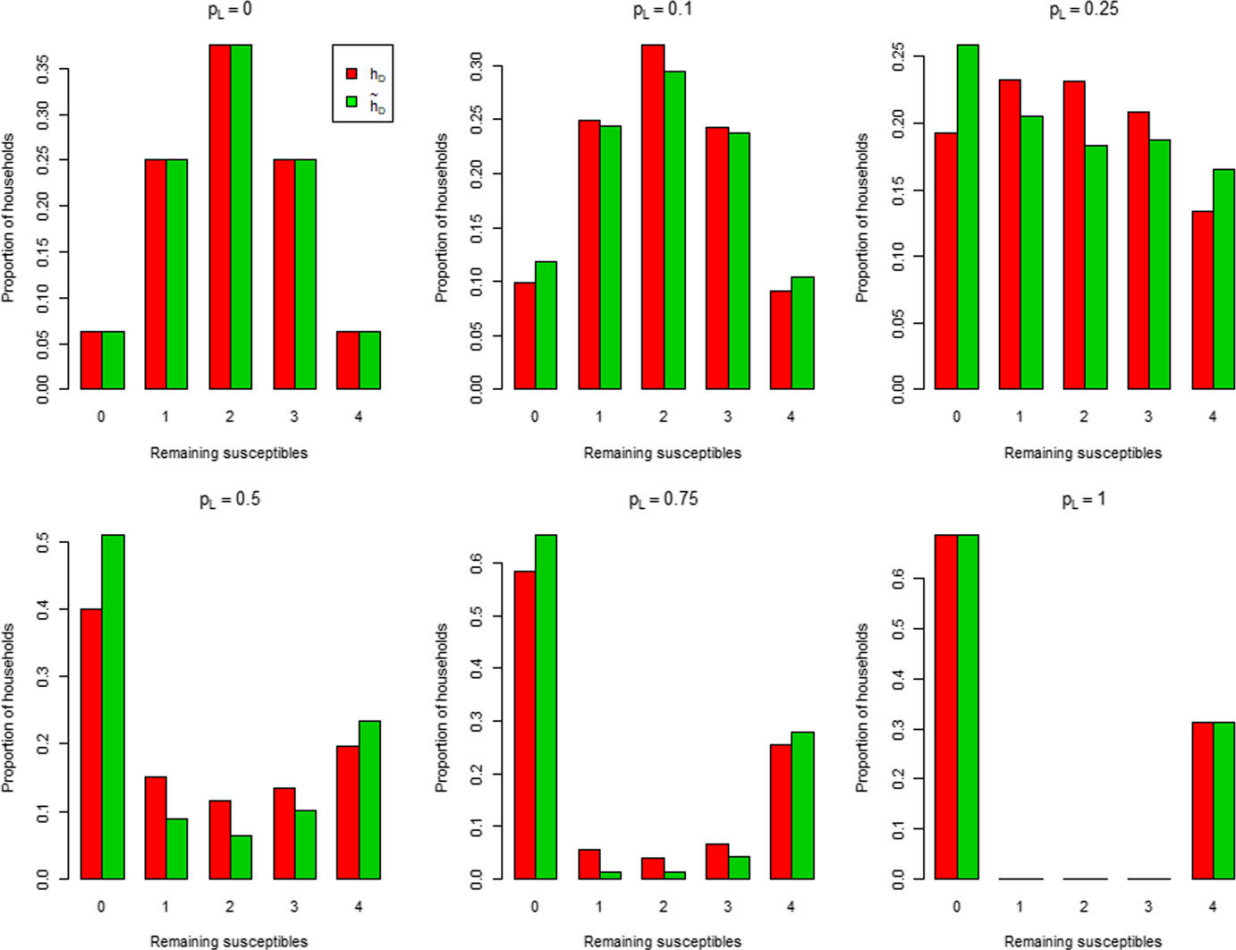
Distribution of the number of susceptibles in a typical household when herd immunity is achieved under $h_D$ and ${\tilde{h}}_D$ when all households have size 4. For each $p_L$, $\lambda _G$ is chosen so that $R_0=2$

#### Accuracy of the approximation of $h_D$ by ${\tilde{h}}_D$

The disparity between $h_D$ and its approximation ${\tilde{h}}_D$ depends on the distribution of susceptibles inside households when herd immunity is achieved. When $\lambda _L=0$, there is no within-household spread; this distribution is the same under $h_D$ as under ${\tilde{h}}_D$. The same conclusion holds when $\lambda _L=\infty$, since all single-household epidemics end immediately (everyone in a contacted household becomes infected as soon as that household is contacted). Thus, when $\lambda _L=0$ or $\lambda _L=\infty$, we have $h_D = {\tilde{h}}_D$. For epidemics with $0< \lambda _L < \infty$, local epidemics are run to termination under ${\tilde{h}}_D$, but not under $h_D$. This, coupled with a lower global infection rate under ${\tilde{h}}_D$, leads to ${\tilde{h}}_D$ being more strongly governed by within-household spread. A consequence of this is the distribution of susceptibles among households being more clumped under ${\tilde{h}}_D$ than $h_D$, leading to a larger proportion of households with no susceptible individuals than under $h_D$, which often results in both ${\tilde{h}}_D > h_D$ and the approximation becoming worse as $n_{\max }$ increases (cf. the discussion following Theorem 4.1 in Sect. 4.2.2). The above-mentioned clumping is illustrated in Fig. 1, which considers the case of a common household size $n=4$, with $p_L$ varying in [0, 1] and $\lambda _G$ being chosen so that $R_0=2$. When $p_L=0$, there is no within-household spread and the distribution of the number of susceptibles in a typical household when herd immunity is reached is $\textrm{Bin} (4, 0.5)$ under both $h_D$ and ${\tilde{h}}_D$. Note the agreement in the two distributions of susceptibles when $p_L=1$. For $p_L \in (0,1)$, the distribution has greater mass at the extremes 0 and 4 under ${\tilde{h}}_D$ than $h_D$. We suspect that ${\tilde{h}}_D > h_D$ holds generally for a common household size $n > 1$ (with $0< \lambda _L < \infty$) and give numerical examples supporting this claim in Sect. 5.1. It is possible (but atypical) for ${\tilde{h}}_D < h_D$ to occur, and the difference is small in the cases we have met. An example is a household structure comprised of households of size 1 and $n > 1$ only; see Fig. 7 in Sect. 5.1.

**Fig. 2:**
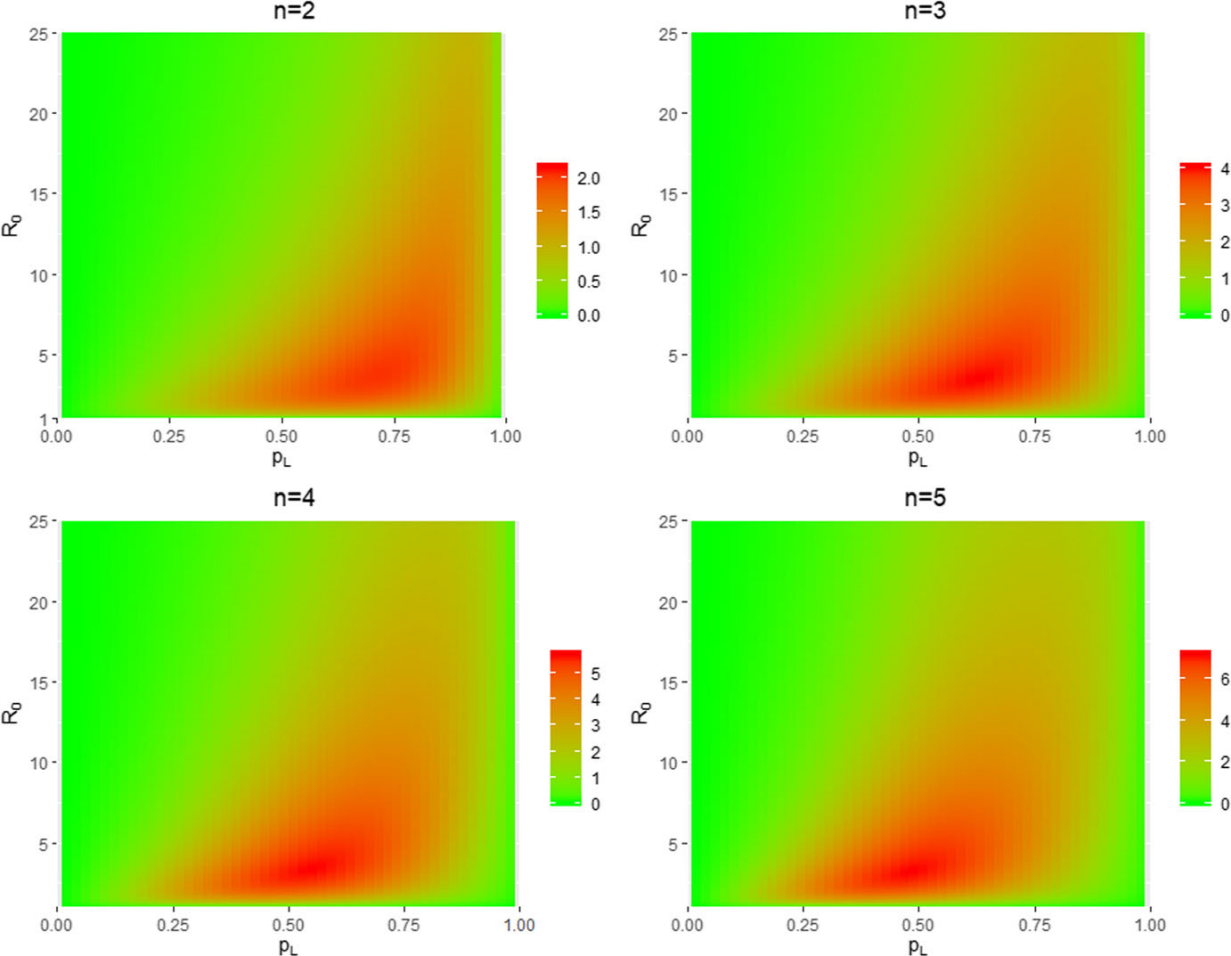
Heat maps of the percentage error $100 \left|{\tilde{h}}_D-h_D \right|/h_D$ as a function of $(p_L, R_0)$ for common household sizes $n=2,3,4$ and 5

The accuracy of the approximation of $h_D$ by ${\tilde{h}}_D$ is explored in Fig. 2, which shows heat maps of the percentage error $100 \left|{\tilde{h}}_D-h_D \right|/h_D$ as a function of $(p_L, R_0)$ for common household sizes $n=2,3,4$ and 5. Observe that the percentage errors are all small, increase with *n* and are greatest for intermediate values of $p_L$. The maximum percentage error as $(p_L, R_0)$ varies over $[0, 1] \times (1, 25]$ for each choice of *n* and for some other household size distributions are given in Table 1. Note that the value of $p_L$ at which this maximum is attained tends to decrease with mean household size $\mu _H=\sum _{n=1}^{\infty } n \alpha _n$. This may be a consequence of the fact that for fixed $p_L$ the fraction infected by a single-household epidemic increases with household size. The maximum percentage errors are small, except for countries with large mean household sizes. Moreover, these are maximum errors and even if they are not small, the error is small for many choices of parameter values, as illustrated by the $n=5$ heat map in Fig. 2. Thus, ${\tilde{h}}_D$ is generally a very good approximation of $h_D$.

**Fig. 3:**
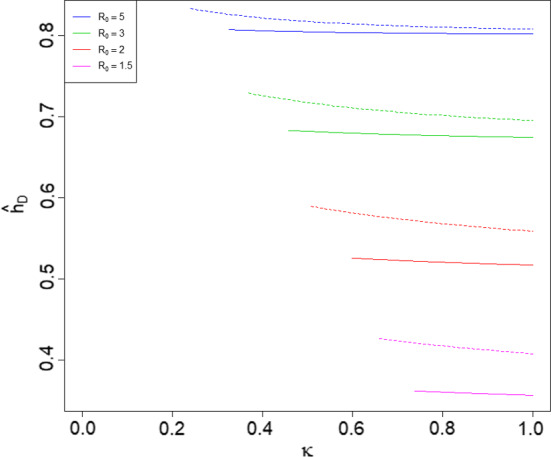
Values of ${\hat{h}}_D$ with global restrictions scaled by a factor $\kappa$ for the duration of the first epidemic, using the UK household size distribution (solid lines) and Morocco’s household size distribution (dashed lines), taking $\lambda _L=0.5$ and considering several values of $R_0$

**Table 1:** The maximum percentage error for ${\tilde{h}}_D$ approximating $h_D$ when $(p_L,R_0) \in [0,1]\times (1,25]$, together with the parameter values for which the maximum is attained

Household	$\mu _H$	Max error ($\%$)	$R_0$	$p_L$	$\lambda _G$	$\lambda _L$
$n=2$	2	2.051	3.711	0.718	3.109	2.547
$n=3$	3	4.019	3.509	0.629	2.555	1.698
$n=4$	4	5.711	3.332	0.551	2.167	1.226
$n=5$	5	7.161	3.179	0.484	1.883	0.939
${\tilde{\alpha }}_1={\tilde{\alpha }}_4=0.5$	1.6	1.269	1.646	0.517	1.057	1.068
Sweden	2.0	1.859	2.135	0.566	1.418	1.306
UK	2.3	2.533	2.401	0.566	1.585	1.306
Argentina	3.3	4.236	2.479	0.495	1.386	0.979
Morocco	4.6	7.367	2.633	0.393	1.293	0.649
Chad	5.8	7.350	2.350	0.343	0.990	0.521
Pakistan	6.8	8.444	2.528	0.338	1.103	0.511

The first four rows correspond to a common household of size *n*. The fifth row corresponds to ${\tilde{\alpha }}_1={\tilde{\alpha }}_4 = 0.5$ and the remaining rows correspond to real-world household size distributions (see Sect. 5.2)

#### Impact of restrictions

Note that the parameter $\kappa$ corresponds to restrictions being placed on the population which affect only the global infection rate; the severity of such restrictions increases as $\kappa$ decreases. Since ${\hat{\kappa }}$ is chosen such that the second epidemic is at criticality, ${\hat{\kappa }}$ corresponds to the most severe restrictions that can be placed for the whole duration of the first epidemic, such that the second epidemic is not supercritical; more severe restrictions will leave the second epidemic supercritical, so herd immunity will not be achieved. Recall that ${\hat{h}}_D$ denotes the disease-induced herd immunity level when restrictions are in place. Numerical investigations suggest that as $\kappa$ increases from ${\hat{\kappa }}$ to 1, ${\hat{h}}_D$ decreases; see Fig. 3. In this example, which uses the UK and Morocco household size distributions (see Sect. 5.2), such restrictions have only a small effect on the herd immunity level ${\hat{h}}_D$, with the effect being larger for Morocco. Moreover, when $\lambda _L$ is fixed, we observe that ${\hat{\kappa }}$ decreases as $R_0$ increases. Repeating these calculations for other values of $\lambda _L$ (not shown) reveals similar patterns and suggests that the effect is largest when $\lambda _L$ is around 0.5. All of these observations regarding the UK and Morocco household size distribution are also seen with larger and more variable household size distributions: the effect of $\kappa$ on ${\hat{h}}_D$ remains small but becomes slightly larger if the household size distribution is more variable, ${\hat{\kappa }}$ decreases with increasing $R_0$ and the effect of varying $\kappa$ seems greatest when $\lambda _L \approx 0.5$.

## Comparison of ${\tilde{h}}_D$ and $h_C$

### Outline

This section presents results concerning orderings of ${\tilde{h}}_D$ and $h_C$. Since both of these quantities depend only on final outcome properties of the epidemic, they are invariant to the distribution of the latent period and we therefore take $T_E=0$, corresponding to the SIR setting, in this section. The problem of solving for ${\tilde{h}}_D$ is not analytically tractable when $0<\lambda _L<\infty$. We hence begin with the highly locally infectious case where $\lambda _L = \infty$, considered in Becker and Dietz (1995), for which a framework for comparison of ${\tilde{h}}_D$ and $h_C$ is established in Sect. 4.2.1 and explicit progress is made. This is then applied to several household size distributions, beginning with all households being the same size, where ${\tilde{h}}_D>h_C$ is established (Theorem 4.1). Further, in Sect. 4.2.2 we show that for a common household size *n*, the maximum of ${\tilde{h}}_D-h_C$ as a function of $\lambda _G$ occurs when $\lambda _G=\frac{4}{(1+n)\textrm{E}[T_I]}$, corresponding to $R_0=2$ (Theorem 4.2). A necessary and sufficient condition for ${\tilde{h}}_D=h_C$ in the highly locally infectious case (Theorem 4.3) is derived in Sect. 4.2.3, leading to study of ${\tilde{h}}_D$ and $h_C$ for epidemics just above criticality (Theorem 4.4 in Sect. 4.2.4), as well as highly locally and globally infectious epidemics (Theorem 4.6 in Sect. 4.2.5). In Sect. 4.2.6 we derive a result for household structures with only households of size 1 and $n>1$ (Theorem 4.7). The weakly locally infectious case $\lambda _L \rightarrow 0$ is treated in Sect. 4.3 and a condition for ${\tilde{h}}_D>h_C$ is derived (Corollary 4.9).

We consider the general case $0<\lambda _L<\infty$, both analytically and numerically, when all households are of the same size. In Sect. 4.4, we prove that ${\tilde{h}}_D>h_C$ for a common household size $n=2$ and $n=3$ (Theorem 4.11). We conjecture that ${\tilde{h}}_D > h_C$ holds for common household size $n \ge 4$; supporting evidence for this conjecture is provided in Sect. 5.1.

### Highly locally infectious case

#### General framework

In the highly locally infectious case ($\lambda _L\rightarrow \infty$) explicit analytical progress is possible, as any infected individual will infect their whole household. We therefore have $\mu _n(\lambda _L)=n$ for $n=0,1,\dots$. Using (2.1), we find that $R_*=\lambda _G \textrm{E}[T_I] \mu _{{\tilde{H}}}$, where $\mu _{{\tilde{H}}}=\textrm{E}[{\tilde{H}}]=\sum _{n=1}^{\infty }n{\tilde{\alpha }}_n$ is the mean size of the household of an individual chosen uniformly at random from the population. Thus, $R_*>1$ if and only if $\lambda _G > \frac{1}{\mu _{{\tilde{H}}}\textrm{E}[T_I]}$. Substituting $\mu _n(\lambda _L)=n$ into (3.1) and solving ${\hat{R}}_U(c)=1$ gives $h_C$ as the unique solution *c* of

4.1 $$\begin{aligned} \sum _{n=1}^{\infty }{\tilde{\alpha }}_n\sum _{v=0}^n\left( {\begin{array}{c}n\\ v\end{array}}\right) c^v\left( 1-c\right) ^{n-v}\frac{(n-v)^2}{n}\lambda _G\textrm{E}[T_I]=1. \end{aligned}$$

The inner sum in (4.1) can be evaluated using the second moment of a $\text{ Bin }(n,1-c)$ random variable. Using the definition of $\mu _{{\tilde{H}}}$, it follows that $h_C$ is given by the unique solution in (0, 1) of the quadratic equation

4.2 $$\begin{aligned} h_C(1-h_C)+\mu _{{\tilde{H}}}(1-h_C)^2-\frac{1}{\lambda _G\textrm{E}[T_I]}=0, \end{aligned}$$

yielding

4.3 $$\begin{aligned} h_C=1-\frac{\sqrt{1+\frac{4(\mu _{{\tilde{H}}}-1)}{\lambda _G\textrm{E}[T_I]}}-1}{2(\mu _{{\tilde{H}}}-1)}. \end{aligned}$$

As noted at the end of Sect. 3.1, $h_C=1-R_0^{-1}$ in the present highly locally infectious case.

Turning to the disease-induced herd immunity level ${\tilde{h}}_D$, consider the first epidemic with global infection rate ${\hat{\kappa }} \lambda _G$, where ${\hat{\kappa }}$ solves ${\hat{R}}_{DI}({\hat{\kappa }})=1$ as described in Sect. 3.2.2. Let $z({\hat{\kappa }})$ be the fraction of the population infected by that epidemic and $\pi =\exp (-{\hat{\kappa }}\lambda _G E[T_I]z({\hat{\kappa }}))$, the probability that any given susceptible avoids global contact during that epidemic. For $n=1,2,\dots$ and $v=0,1,\dots ,n$, let $x_{n,v}(\pi )$ be the proportion of households with *n* members which have *v* infected in this epidemic and thus immune to the second epidemic.

Letting $R_{DI}(\pi ) = {\hat{R}}_{DI}({\hat{\kappa }})$, we have

4.4 $$\begin{aligned} R_{DI}(\pi )=\sum _{n=1}^{\infty }{\tilde{\alpha }}_n\sum _{v=0}^nx_{n,v}(\pi )\frac{(n-v)^2}{n}\lambda _G\textrm{E}[T_I]. \end{aligned}$$

In the highly locally infectious case, a given individual escapes infection if and only if their whole household avoids global infection. Thus $x_{n,0}(\pi )=\pi ^n$, $x_{n,n}(\pi )=1-\pi ^n$, and $x_{n,v}(\pi )=0$ for $v \notin \{0,n\}$. Substitution into (4.4) yields

4.5 $$\begin{aligned} R_{DI}(\pi ) = \sum _{n=1}^{\infty }n{\tilde{\alpha }}_n\pi ^n\lambda _G\textrm{E}[T_I]. \end{aligned}$$

Note that ${\tilde{\mu }}_n(\infty ,\pi ) = n(1-\pi ^n)$, so equation (2.4) implies that the final proportion infected in the first epidemic is ${\tilde{h}}_D = z({\hat{\kappa }}) = 1-\sum _{n=1}^{\infty }{\tilde{\alpha }}_n\pi ^n$. Thus,

4.6 $$\begin{aligned} {\tilde{h}}_D=1-f_{{\tilde{H}}}(\pi ), \end{aligned}$$

where $f_{{\tilde{H}}}(\pi )=\sum _{n=1}^{\infty }{\tilde{\alpha }}_n\pi ^n$ is the probability-generating function of ${\tilde{H}}$. Setting $R_{DI}(\pi )=1$ in (4.5) yields

4.7 $$\begin{aligned} \pi f^{\prime }_{{\tilde{H}}}(\pi )=\frac{1}{\lambda _G\textrm{E}[T_I]}. \end{aligned}$$

Combined with (4.2), we have a framework to compare ${\tilde{h}}_D$ and $h_C$. Note, however, that the system given by (4.6) and (4.7) does not always allow closed-form calculation of ${\tilde{h}}_D$.

Note that in this subsection dealing with the highly local infectious case, the distribution of $T_I$ only enters our results through its mean $E[T_I]$.

#### Common household size

Suppose that all households are of size *n*. When $n=1$ the model reduces to the standard homogeneously mixing model, so ${\tilde{h}}_D = h_C = 1 - R_0^{-1}$. Therefore assume that $n>1$. Using (4.3),

4.8 $$\begin{aligned} h_C=1-\frac{\sqrt{1+\frac{4(n-1)}{\lambda _G \textrm{E}[T_I]}}-1}{2(n-1)}. \end{aligned}$$

Note that $f_{{\tilde{H}}}(\pi ) = \pi ^n$, so (4.6) and (4.7) yield

4.9 $$\begin{aligned} {\tilde{h}}_D=1-\frac{1}{n\lambda _G\textrm{E}[T_I]}. \end{aligned}$$

##### Theorem 4.1

Consider the highly locally infectious case with common household size $n>1$. Then ${\tilde{h}}_D>h_C$ if $R_*>1$.

##### Proof

The claim is established by subtracting equation (4.8) from (4.9), and letting $x=\frac{1}{\lambda _G \textrm{E}[T_I]}$ for ease of exposition, so $R_*=n\lambda _G\textrm{E}[T_I] =\frac{n}{x}$. Expressing explicitly the dependence of ${\tilde{h}}_D$ and $h_C$ on *x*, we obtain

4.10 $$\begin{aligned} {\tilde{h}}_D(x)-h_C(x)= & {} \frac{\sqrt{1+4x(n-1)}-1}{2(n-1)} -\frac{x}{n}=\frac{n\sqrt{1+4x(n-1)}-n-2x(n-1)}{2n(n-1)}.\nonumber \\ \end{aligned}$$

The result then follows by elementary manipulations of (4.10), since $R_*>1$ implies $x \in (0, n)$. $\square$

A heuristic justification for Theorem 4.1 is as follows. In disease-induced herd immunity, after the first epidemic, households contain either 0 or *n* susceptibles, depending on whether that household was infected. Consider a randomly chosen individual contacted globally in the second epidemic. If this individual is immune, this contact contributes no further infection. Otherwise, they begin an epidemic within their household which, in the highly locally infectious case, will infect all non-immune members. Hence, under disease-induced herd immunity, the potential for within-spread is as high as possible (the rest of the household is susceptible). Thus disease-induced herd immunity corresponds to the worst possible vaccination strategy for a given coverage, resulting in ${\tilde{h}}_D>h_C$.

A further result provides a link between the highly locally infectious case and $R_0$ for the households model, in which we treat ${\tilde{h}}_D-h_C$ as a function of $\lambda _G$.

##### Theorem 4.2

Under the same assumptions as Theorem 4.1, ${\tilde{h}}_D-h_C$ has a unique maximum as a function of $\lambda _G$ which is attained when $\lambda _G=\frac{4}{(1+n)\textrm{E}[T_I]}$, corresponding to $R_0=2$.

##### Proof

We show that ${\tilde{h}}_D(x)-h_C(x)$ has a unique stationary point, which must be a maximum since, from (4.10), ${\tilde{h}}_D(x)-h_C(x) \rightarrow 0$ as $x\downarrow 0$ and $x\uparrow n$, corresponding to $R_*\rightarrow \infty$ and $R_*\rightarrow 1$, respectively, and by Theorem 4.1, ${\tilde{h}}_D(x)-h_C(x)>0$ for $x \in (0, n)$. Then we find the value of *x* which yields the maximum and compute the corresponding $R_0$ value.

Ignoring the denominator in (4.10), differentiation with respect to *x* and equating to 0 leads us to solve

$$\begin{aligned} \frac{4n(n-1)}{2\sqrt{1+4x(n-1)}}-2(n-1)=0 \Longleftrightarrow {\hat{x}}=\frac{n+1}{4} \Longleftrightarrow {\hat{\lambda }}_G=\frac{4}{(1+n)\textrm{E}[T_I]}. \end{aligned}$$

In the highly locally infectious case, all secondary infections in a household are attributed to the primary case, so $\mu _0^{(k)}=1$, $\mu _1^{(k)}=k-1$ and $\mu _j^{(k)}=0$ for $j \notin \{0,1\}$. Setting $\lambda _G={\hat{\lambda }}_G$ in (2.3) gives that $R_0$ is the unique positive root of $g_n(\lambda )=0$, where

$$\begin{aligned} g_n(\lambda )=1-\frac{4}{1+n}\left( \frac{1}{\lambda }+\frac{n-1}{\lambda ^2} \right) . \end{aligned}$$

Now $g_n(2)=0$, so the maximum of ${\tilde{h}}_D-h_C$ is attained when $R_0=2$, as claimed. $\square$

#### Necessary and sufficient condition for ${\tilde{h}}_D = h_C$ for all $\lambda _G$

The framework given in Sect. 4.2.1 for the highly locally infectious case enables proof of the following result. For $\theta \in (0,1]$, we write ${\tilde{H}} \sim \textrm{Geom}(\theta )$ if ${\tilde{H}}$ has a geometric distribution with probability mass function

$$\begin{aligned} \textrm{P}({\tilde{H}}=x)=\theta (1-\theta )^{x-1} \quad (x=1,2\dots ). \end{aligned}$$

##### Theorem 4.3

In the highly locally infectious case, ${\tilde{h}}_D = h_C$ for all $\lambda _G$ such that $R_*>1$ if and only if ${\tilde{H}} \sim \textrm{Geom}(\mu _{{\tilde{H}}}^{-1})$.

##### Proof

Recall that $R_*>1$ if and only if $\lambda _G > \frac{1}{\mu _{{\tilde{H}}}\textrm{E}[T_I]}$. We begin by assuming ${\tilde{h}}_D-h_C=0$ for all $\lambda _G > \frac{1}{\mu _{{\tilde{H}}}\textrm{E}[T_I]}$ and solving for ${\tilde{H}}$. Equations (4.2) and (4.6) give

$$\begin{aligned} f_{{\tilde{H}}}(\pi )(1-f_{{\tilde{H}}}(\pi ))+\mu _{{\tilde{H}}}(f_{{\tilde{H}}}(\pi ))^2=\frac{1}{\lambda _G\textrm{E}[T_I]}. \end{aligned}$$

Equation (4.7) implies that

4.11 $$\begin{aligned} G(\pi ):=f_{{\tilde{H}}}(\pi )(1-f_{{\tilde{H}}}(\pi ))+\mu _{{\tilde{H}}}(f_{{\tilde{H}}}(\pi ))^2-\pi f^{\prime }_{{\tilde{H}}}(\pi ) =0\;\;\;\;\; (\pi \in [0,1)).\nonumber \\ \end{aligned}$$

Further, $f_{{\tilde{H}}}(1)=1$ since $f_{{\tilde{H}}}(\pi )$ is a probability-generating function. This separable ODE can be solved to yield, for $0\le \pi \le 1$,

4.12 $$\begin{aligned} f_{{\tilde{H}}}(\pi )=\frac{\pi \mu _{{\tilde{H}}}^{-1}}{1-\pi \left( 1-\mu _{{\tilde{H}}}^{-1}\right) }, \end{aligned}$$

which is precisely the probability-generating function of a $\textrm{Geom}(\mu _{{\tilde{H}}}^{-1})$ random variable. This establishes the only if part of the equivalence claim. For the converse, assume that ${\tilde{H}}$ follows a geometric distribution with parameter $\mu _{{\tilde{H}}}$. Then (4.11) holds and the logic for the above proof is reversible thereafter, so the result follows. $\square$

When ${\tilde{H}}$ follows a geometric distribution, *H* follows a logarithmic distribution. Note that real-life household size distributions will have a finite maximum size, so for any realistic household size distribution ${\tilde{h}}_D=h_C$ will not hold for all $\lambda _G$ in the highly locally infectious case. We typically observe ${\tilde{h}}_D>h_C$ for real-life household size distributions and comment upon this further in Sect. 5.1.

#### Just supercritical epidemics

We can use the framework provided in Sect. 4.2.1 to give an ordering of ${\tilde{h}}_D$ and $h_C$ for epidemics which are just above threshold, i.e. when $R_*$ is just above 1, so $\pi$ is just below 1. We hence establish an ordering of ${\tilde{h}}_D$ and $h_C$ by considering $G(\pi )$, given in (4.11), in the neighbourhood of $\pi =1$. Assuming a fraction $z(\pi )$ of individuals are infected in the first epidemic gives a threshold parameter $R_{DI}(\pi )$ for the second epidemic, as given in (4.4). Vaccinating the same proportion uniformly at random gives a threshold parameter $R_U(\pi ) = {\hat{R}}_U(z(\pi ))$. Here, $R_U(\pi )-R_{DI}(\pi )=\lambda _G \textrm{E}[T_I]G(\pi )$. Clearly we have $G(1)=0$, since $f_{{\tilde{H}}}(1)=1$ and $f^{\prime }_{{\tilde{H}}}(1)=\mu _{{\tilde{H}}}$. Let $G^{(k)}$ be the $k^{\textrm{th}}$ derivative of *G* and define

4.13 $$\begin{aligned} k^{*}=\inf _{k\ge 1}\{k: G^{(k)}(1)\ne 0\}. \end{aligned}$$

Suppose that $G^{(k^*)}(1) > 0$. Then $G(\pi )<0$ for $\pi$ just below 1, so $R_U(\pi )<R_{DI}(\pi )$ for such $\pi$. Hence, $R_U(\pi )<1$ if $R_{DI}(\pi )=1$ and it follows that ${\tilde{h}}_D>h_C$ for epidemics which are just above threshold. A similar argument shows that ${\tilde{h}}_D<h_C$ if $G^{(k^*)}(1) < 0$. If ${\tilde{H}}$ follows a geometric distribution then $G^{(k)}(1)=0$ for all $k\ge 1$, otherwise ${\tilde{h}}_D \ne h_C$. Determining the ordering of ${\tilde{h}}_D$ and $h_C$ reduces to comparing factorial moments of ${\tilde{H}}$ to those of a geometric distribution with parameter $\mu _{{\tilde{H}}}^{-1}$. For a random variable ${\tilde{H}}$ define, for $i=1,2,\dots$, the factorial moments $\mu _{{\tilde{H}}}^{[i]}=\textrm{E}[{\tilde{H}}({\tilde{H}}-1)\dots ({\tilde{H}}-i+1)]$, with $\mu _{{\tilde{H}}}^{[0]}=1$. Note that $\mu _{{\tilde{H}}}^{[1]}=\mu _{{\tilde{H}}}$.

##### Theorem 4.4

Let ${\tilde{H}}$ be a given size-biased household size distribution with mean $\mu _{{\tilde{H}}}$ and factorial moments $\mu _{{\tilde{H}}}^{[i]}$ $(i=0,1,\dots )$. Suppose that $l^{*}=\displaystyle \inf _{k \ge 2}\{k: \mu _{{\tilde{H}}}^{[k]} \ne k!\mu _{{\tilde{H}}}( \mu _{{\tilde{H}}}-1)^{k-1}\} < \infty$. Then ${\tilde{h}}_D>h_C$ for highly locally infectious epidemics which are just above threshold if $\mu _{{\tilde{H}}}^{[l^*]} < l^*! \mu _{{\tilde{H}}}(\mu _{{\tilde{H}}}-1)^{l^*-1}$, otherwise ${\tilde{h}}_D<h_C$ for such epidemics.

##### Proof

For $i=0,1,2 \dots$, let $\hat{{\mu }}_{{\tilde{H}}}^{[i]}$ be the $i^{th}$ factorial moment of ${\tilde{H}}$ when ${\tilde{H}} \sim \textrm{Geom}(\mu _{{\tilde{H}}}^{-1})$. Then $\hat{{\mu }}_{{\tilde{H}}}^{[0]}=1$ and

$$\begin{aligned} {\hat{\mu }}^{[n]}_{{\tilde{H}}}=n!\mu _{{\tilde{H}}}\left( \mu _{{\tilde{H}}}-1\right) ^{n-1}\;\;\;\;\; (n=1,2,\dots ). \end{aligned}$$

We compute $G^{(k)}(1)$ using the general Leibniz rule. For $k = 1,2,\dots$ we have

4.14 $$\begin{aligned} G^{(k)}(\pi )= & {} (1-k)f_{{\tilde{H}}}^{(k)}(\pi )+(\mu _{{\tilde{H}}}-1)\nonumber \\ {}{} & {} \times \sum _{i=0}^k\left( {\begin{array}{c}k\\ i\end{array}}\right) f_{{\tilde{H}}}^{(i)}(\pi )f_{{\tilde{H}}}^{(k-i)}(\pi )-\pi f_{{\tilde{H}}}^{(k+1)}(\pi ), \end{aligned}$$

leading to

4.15 $$\begin{aligned} G^{(k)}(1)=(1-k)\mu _{{\tilde{H}}}^{[k]}+(\mu _{{\tilde{H}}}-1)\sum _{i=0}^k\left( {\begin{array}{c}k\\ i\end{array}}\right) \mu _{{\tilde{H}}}^{[k-i]}\mu _{{\tilde{H}}}^{[i]}-\mu _{{\tilde{H}}}^{[k+1]}. \end{aligned}$$

Note that $G^{(k)}(1)\equiv \delta _n-\mu ^{[k+1]}_{{\tilde{H}}}$, where $\delta _k$ depends only on $\mu ^{[0]}_{{\tilde{H}}},\mu ^{[1]}_{{\tilde{H}}},\dots ,\mu ^{[k]}_{{\tilde{H}}}$. Recall that $G^{(k)}(1)=0$ for all $k =0,1,\dots$ when ${\tilde{H}} \sim \textrm{Geom}(\mu _{{\tilde{H}}}^{-1})$. Now suppose that $l^{*} < \infty$ and $\mu _{{\tilde{H}}}^{[l^*]} < l^*! \mu _{{\tilde{H}}}(\mu _{{\tilde{H}}}-1)^{l^*-1}$. Then $G^{(k)}(1)=0$ for $k=0,1,\dots l^{*}-1$, since $\mu _{{\tilde{H}}}^{[k]}=\hat{{\mu }}_{{\tilde{H}}}^{[k]}$ for $k=0,1,\dots , l^{*}-1$, and $G^{(l^{*})}(1)>0$ since $\mu _{{\tilde{H}}}^{[l^{*}]}<\hat{{\mu }}_{{\tilde{H}}}^{[l^{*}]}$. Hence, ${\tilde{h}}_D>h_C$ by the observation following (4.13). A similar argument holds when $\mu _{{\tilde{H}}}^{[l^*]} > l^*! \mu _{{\tilde{H}}}(\mu _{{\tilde{H}}}-1)^{l^*-1}$.

$$\square $$

In many cases only the first derivative of *G* is required. The following corollary, which involves only the mean and variance of ${\tilde{H}}$, is an immediate consequence of Theorem 4.4 in the case $l^{*}=2$.

##### Corollary 4.5

If $\textrm{var}({\tilde{H}})<\textrm{E}[{\tilde{H}}]\textrm{E}[{\tilde{H}}-1]$ then ${\tilde{h}}_D>h_C$ for highly locally infectious epidemics which are just above threshold. If $\textrm{var}({\tilde{H}})>\textrm{E}[{\tilde{H}}]\textrm{E}[{\tilde{H}}-1]$ then ${\tilde{h}}_D<h_C$ for highly locally infectious epidemics which are just above threshold.

#### Highly locally and globally infectious epidemics

The framework in Sect. 4.2.1 can also be used to consider highly locally and highly globally infectious epidemics. This corresponds to the case where $\pi$, the global escape probability, is small. Considering $\pi \downarrow 0$ yields the following theorem. (Recall that, for $n=1,2,\dots$, ${\tilde{\alpha }}_n = \textrm{P}({\tilde{H}}=n)$.)

##### Theorem 4.6

Suppose that $n^{*}=\inf _{n\ge 2}\{n: G^{(n)}(0)\ne 0\} < \infty$. Then ${\tilde{h}}_D>h_C$ for all sufficiently small $\pi >0$ if ${\tilde{\alpha }}_{n^{*}}<{\tilde{\alpha }}_1^{n^{*}}(\mu _{{\tilde{H}}}-1)^{n^{*}-1}$, otherwise ${\tilde{h}}_D<h_C$ for such $\pi$.

##### Proof

Note that, for $i=0,1,\dots$, we have $f^{(i)}_{{\tilde{H}}}(0)=i!{\tilde{\alpha }}_i$, with ${\tilde{\alpha }}_0=0$. Substituting $\pi =0$ into (4.14) yields, after elementary algebra, that $G^{(1)}(0)=0$ and, for $n\ge 2$,

4.16 $$\begin{aligned} G^{(n)}(0)= n!\left( (1-n){\tilde{\alpha }}_n+(\mu _{{\tilde{H}}}-1)\sum _{k=1}^{n-1}{\tilde{\alpha }}_{n-k}{\tilde{\alpha }}_k \right) . \end{aligned}$$

Suppose that $G^{(n)}(0)=0$ for $n=1,2,\dots$. Iterating (4.16) gives ${\tilde{\alpha }}_n={\tilde{\alpha }}_1^n(\mu _{{\tilde{H}}}-1)^{n-1}$. Then ${\tilde{\alpha }}_1=\mu _{{\tilde{H}}}^{-1}$ (since $\sum _{n=1}^{\infty }{\tilde{\alpha }}_n=1$), so ${\tilde{H}} \sim \textrm{Geom}(\mu _{{\tilde{H}}}^{-1})$. With $n^{*}$ as in the statement of the theorem, we have that $G^{(n^{*})}(0)>0$ implies ${\tilde{h}}_D<h_C$, and $G^{(n^{*})}(0)<0$ implies ${\tilde{h}}_D>h_C$, from which the result follows.

$$\square $$

Similarly to the just supercritical case, the only distribution for ${\tilde{H}}$ which has $G^{(n)}(0)=0$ for all *n* is the geometric distribution with parameter $\mu _{{\tilde{H}}}^{-1}$. Theorem 4.6 then reduces the ordering of ${\tilde{h}}_D$ and $h_C$ to iterative comparison of the probability mass function of ${\tilde{H}}$ with the relevant geometric distribution.

**Fig. 4:**
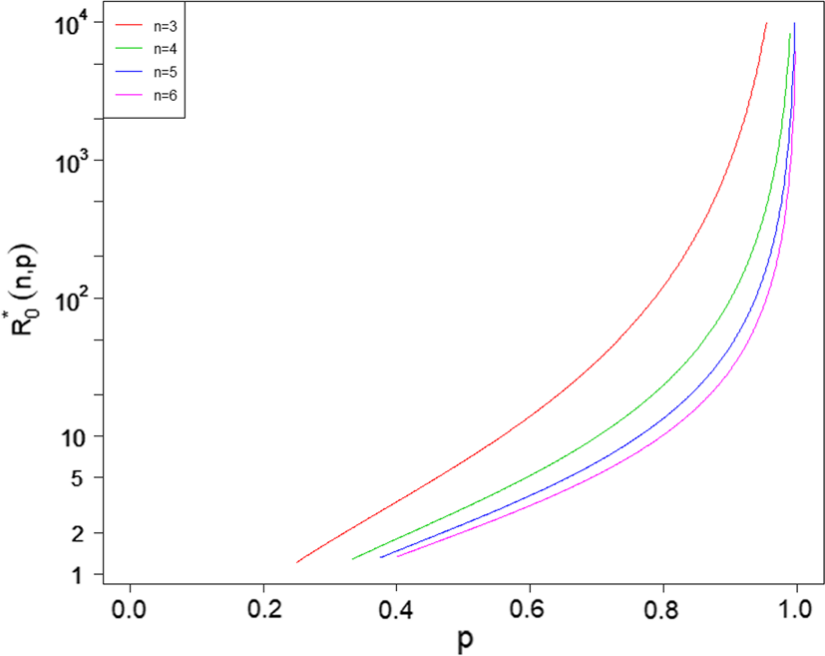
The value of $R_0^*(n,p)$ (on a logarithmic scale) as a function of *p* for $n \in \{3,4,5,6\}$

#### Households of size 1 and $n>1$

Theorem 4.1 in Sect. 4.2.2 shows that, in the highly locally infectious case, ${\tilde{h}}_D>h_C$ for all $\lambda _G$ when the households all have the same size. We now consider the simplest setting when there is variability in household size, i.e. the case where there are only two household sizes, 1 and $n>1$. For $0< p <1$, let *p* denote the proportion of individuals who belong to a household of size *n*. Thus ${\tilde{\alpha }}_n = p$ and ${\tilde{\alpha }}_1 = 1-p$. We consider how ${\tilde{h}}_D - h_C$ varies with *p*, with a view towards obtaining different orderings of ${\tilde{h}}_D$ and $h_C$ as the household structure changes. The following theorem, proved in Appendix B.1, shows that the ordering of ${\tilde{h}}_D$ and $h_C$ is less straightforward when household size is variable.

##### Theorem 4.7

Suppose that ${\tilde{\alpha }}_n = p = 1-{\tilde{\alpha }}_1$, where $n>1$ and $0<p<1$, and $R_*>1$. If $n=2$, then ${\tilde{h}}_D>h_C$ for all *p*. For $n\ge 3$, if $p \le \frac{n-2}{2(n-1)}$ then ${\tilde{h}}_D<h_C$ for all $\lambda _G$. If $p>\frac{n-2}{2(n-1)}$ then there exists $\lambda _G^{*}(n,p)$ such that ${\tilde{h}}_D>h_C$ for $\lambda _G<\lambda _G^{*}(n,p)$ and ${\tilde{h}}_D<h_C$ for $\lambda _G>\lambda _G^{*}(n,p)$. Further, $\lambda _G^{*}(n,p)=\left[ \textrm{E}[T_I]{\hat{\pi }}_n(p)\left( 1-p+np{{\hat{\pi }}_n(p)}^{n-1}\right) \right] ^{-1}$, where ${\hat{\pi }}_n(p)$ is the unique root in (0, 1) of

4.17 $$\begin{aligned} \pi ^{\frac{n}{2}-1}-p\pi ^{n-1}-(1-p)=0. \end{aligned}$$

One can solve (4.17) for ${\hat{\pi }}$ numerically and thereby determine $\lambda _G^{*}(n,p)$, and the corresponding value $R_0^*(n,p)$ of $R_0$ (which is independent of $E[T_I]$), such that the change in the ordering of ${\tilde{h}}_D$ and $h_C$ occurs. Figure 4 shows $R_0^*(n,p)$ as a function of *p* for various *n*, with ${\tilde{h}}_D<h_C$ above the plotted line and ${\tilde{h}}_D>h_C$ below it. If $p \le (n-2)/2(n-1)$ then no change of sign occurs and ${\tilde{h}}_D>h_C$ for all values of $R_0>1$.

We see immediately that $R_0^*(n,p)$ decreases with *n* and increases with *p*. One can also show that, for fixed *n*, $\lim _{p\uparrow 1} R_0^*(n,p) = \infty$. Further, $\textrm{var}({\tilde{H}})=(n-1)^2p(1-p)$, so the variability in household size is small when *p* is close to one. Thus Theorem 4.7 shows that, even with low variability in household size, we can have ${\tilde{h}}_D<h_C$; however, in this example, $R_0$ has to be unrealistically large for this to happen.

### Weakly locally infectious case

Consider now the case when the extra local infection is small but non-zero, corresponding to $\lambda _L\rightarrow 0$, with $R_*>1$, $\textrm{E}[T_I]=1$ and $\textrm{var}(T_I)<\infty$. Assume also that $n_{\max }<\infty$.

#### Theorem 4.8

Let $\pi (0)= \frac{1}{\lambda _G}$. We have

$$\begin{aligned} {\tilde{h}}_D(\lambda _L)-h_C(\lambda _L)=2\lambda _L^2\pi (0)^2(1-\pi (0))\left[ \textrm{E}[{\tilde{H}}-1]-\textrm{var}({\tilde{H}})\right] +o(\lambda _L^2). \end{aligned}$$

The proof of Theorem 4.8 involves computing the first three terms of the Maclaurin expansion of ${\tilde{h}}_D(\lambda _L)-h_C(\lambda _L)$ and is given in Appendix B.2, where the assumption that $n_{\max }< \infty$ is also explained. The assumption that $\textrm{E}[T_I]=1$ involves no loss of generality (since time can be rescaled appropriately) and is made to simplify the presentation of the proof. The assumption $\textrm{var}(T_I)<\infty$ is required for the third term in the above-mentioned Maclaurin expansion. Note that when $\lambda _L=0$, $R_* = R_0 = \lambda _G$, whence $h_C = {\tilde{h}}_D = 1-\pi (0)$. The following corollary is an immediate consequence of Theorem 4.8.

#### Corollary 4.9

If $\textrm{var}({\tilde{H}}) < \textrm{E}[{\tilde{H}}-1]$ then ${\tilde{h}}_D>h_C$ for all sufficiently small $\lambda _L>0$. If $\textrm{var}({\tilde{H}}) > \textrm{E}[{\tilde{H}}-1]$ then ${\tilde{h}}_D<h_C$ for all sufficiently small $\lambda _L>0$.

If $\textrm{var}({\tilde{H}}) = \textrm{E}[{\tilde{H}}-1]$, higher terms in the Maclaurin expansion of ${\tilde{h}}_D(\lambda _L)-h_C(\lambda _L)$ are required in order to give an ordering. Note that Corollaries 4.5 and 4.9 produce contrasting orderings of ${\tilde{h}}_D$ and $h_C$ if $\textrm{E}[{\tilde{H}}]-1< \textrm{var}({\tilde{H}}) < \textrm{E}[{\tilde{H}}](\textrm{E}[{\tilde{H}}]-1)$. (See Sect. 5.2 for a numerical exploration of this.)

A result for a common household size $n>1$ follows immediately from Corollary 4.9.

#### Corollary 4.10

Suppose all households are the same size $n>1$. For all sufficiently small $\lambda _L>0$, we have ${\tilde{h}}_D>h_C$.

#### Proof

When the common household size is $n>1$, we have $\textrm{var}({\tilde{H}})=0$ and $\textrm{E}[{\tilde{H}}-1]>0$. Applying Corollary 4.9 then establishes the claim. $\square$

### Common household size with $0<\lambda _L<\infty$

We have shown that, for a common household size *n* and any $\lambda _G$ such that $R_*>1$, we have ${\tilde{h}}_D>h_C$ when $\lambda _L\rightarrow 0$ and $\lambda _L = \infty$. The following theorem considers $\lambda _L \in (0,\infty )$.

#### Theorem 4.11

For a common household size $n=2$ or $n=3$, and for any $\lambda _G$ and $\lambda _L>0$ such that $R_*>1$, we have ${\tilde{h}}_D>h_C$.

#### Proof

Suppose a fraction *z* of the population is infected by a first epidemic in the households model with the above parameters. This leads to a threshold parameter ${\hat{R}}_{DI}(z)$ for the second epidemic. Further, assuming the same proportion are vaccinated uniformly at random gives threshold parameter ${\hat{R}}_U(z)$. We show that ${\hat{R}}_{DI}(z)-{\hat{R}}_U(z)>0$, from which ${\tilde{h}}_D>h_C$ is immediate.

Let $P_i^D$ ($i=0,1,\dots ,n$) be the proportion of households with *i* members immune owing to the first epidemic and let $P_i^U$ ($i=0,1,\dots ,n$) be the analogous quantity with uniformly at random vaccination, both assuming a fraction *z* of the population is immune. Then we can write

$$\begin{aligned} {\hat{R}}_U(z)=\lambda _G\textrm{E}[T_I]\sum _{v=0}^n\left( 1-\frac{v}{n} \right) P_v^U\mu _{n-v}(\lambda _L) \end{aligned}$$

and

$$\begin{aligned} {\hat{R}}_{DI}(z)=\lambda _G\textrm{E}[T_I]\sum _{v=0}^n\left( 1-\frac{v}{n} \right) P_v^D\mu _{n-v}(\lambda _L). \end{aligned}$$

Assuming $n=2$ and considering the proportion of susceptibles remaining after vaccination also yields

$$\begin{aligned} 2P_0^D+P_1^D=2P_0^U+P_1^U. \end{aligned}$$

Now $A:=P_0^D-P_0^U=\pi ^2-(1-z)^2>0$, since the probability an individual avoids global infection $\pi$ is larger than the overall probability it avoids infection $1-z$. We then find

$$\begin{aligned} \begin{aligned} {\hat{R}}_{DI}(z)-{\hat{R}}_U(z)&= \lambda _G\textrm{E}[T_I]\left[ \left( P_0^D-P_0^U\right) \mu _2(\lambda _L)+\frac{1}{2}(P_1^D-P_1^U)\mu _1(\lambda _L)\right] \\&= A\lambda _G\textrm{E}[T_I]\left[ \mu _2(\lambda _L)-\mu _1(\lambda _L)\right] >0,\\ \end{aligned} \end{aligned}$$

since $\mu _2(\lambda _L)>\mu _1(\lambda _L)$ and $A>0$. The result follows and the claim is established for $n=2$. The proof for $n=3$ uses a similar (but more involved) argument and is deferred to Appendix B.3. $\square$

A proof for $n>3$ has not been forthcoming, but we make the following conjecture, which is supported by numerical evidence (Fig. 6) in Sect. 5.1.

#### Conjecture 4.12

For any common household size $n>1$, and for any $\lambda _G$ and $\lambda _L$ such that $R_*>1$, we have ${\tilde{h}}_D>h_C$.

**Fig. 5:**
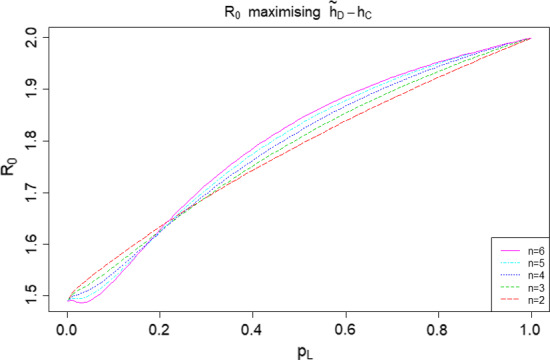
Plot of $R_0$ such that ${\tilde{h}}_D-h_C$ is maximised, where the common household size $n \in \{2,3,4,5,6\}$ and $T_I \sim \textrm{Exp}(1)$

In Theorem 4.2 we show that for a common household size in the highly locally infectious case, the difference ${\tilde{h}}_D-h_C$ is maximised at $R_0=2$. We show numerically that this does not hold when $\lambda _L<\infty$. For ease of visualisation we work in terms of the probability that an infectious individual makes local infectious contact with a given individual in their household, $p_L = p_L(\lambda _L) = 1-\phi (\lambda _L)$, instead of $\lambda _L$ directly. Note that $p_L$ is a monotonic function of $\lambda _L$, with $p_L(0)=0$ and $p_L(\infty )=1$. Taking $T_I \sim \textrm{Exp}(1)$, we have $p_L = \lambda _L(1+\lambda _L)^{-1}$. In Fig. 5 we fix $n \in \{2,3,4,5,6\}$ and $p_L(\lambda _L)$, determine ${\hat{\lambda }}_G = \displaystyle \mathop {\mathrm {arg\,max}}\limits _{\lambda _G}({\tilde{h}}_D-h_C)$, the global infection rate which maximises the difference (assumed to be positive on the basis of Conjecture 4.12) between the herd immunity levels, then calculate the resulting value of $R_0$.

We see that the ‘optimal’ value of $R_0$ broadly increases with $p_L$ and tends to 2 as $p_L \rightarrow 1$, consistent with Theorem 4.2. The dip near $p_L=0$ becomes more pronounced as *n* increases. Similar behaviour occurs for other choices of infectious period distribution.

When $p_L=0$ we have ${\tilde{h}}_D=h_C$, so any value of $\lambda _G$ maximises ${\tilde{h}}_D-h_C$. As such, the value of $R_0$ when $\lambda _L= 0$ is not well-defined, leading to instability when solving numerically. However, in the general setting with variable household sizes we can proceed analytically using Theorem 4.8. We have, as $\lambda _L \downarrow 0$,

4.18 $$\begin{aligned} {\tilde{h}}_D(\lambda _L)-h_C(\lambda _L)= 2\lambda _L^2\pi (0)^2(1-\pi (0))\left[ \textrm{E}[{\tilde{H}}-1]-\textrm{var}({\tilde{H}})\right] +o(\lambda _L^2),\nonumber \\ \end{aligned}$$

where $\pi (0)=\frac{1}{\lambda _G}$. If $\textrm{E}[{\tilde{H}}-1]>\textrm{var}({\tilde{H}})$ ($\textrm{E}[{\tilde{H}}-1]<\textrm{var}({\tilde{H}})$), the right-hand-side of (4.18), ignoring the $o(\lambda _L^2)$ term, is maximised (minimised) at ${\hat{\pi }}_0=\frac{2}{3}$, yielding ${\hat{\lambda }}_G=1.5$. The value of $R_0$ maximising $\left|{\tilde{h}}_D(\lambda _L)-h_C(\lambda _L) \right|$ then satisfies $R_0 \rightarrow 1.5$ as $\lambda _L \rightarrow 0$.

## Numerical comparisons of herd immunity levels

We begin in Sect. 5.1 by numerically illustrating some of the results from Sect. 4, then in Sect. 5.2 we explore how our findings play out in the context of realistic household size distributions. Throughout this section, we restrict attention to the Markovian case, i.e. we assume that $T_I \sim \textrm{Exp}(\gamma )$, and $T_E \sim \textrm{Exp}(\delta )$ if a latent period is present.

### Illustrative examples

**Fig. 6:**
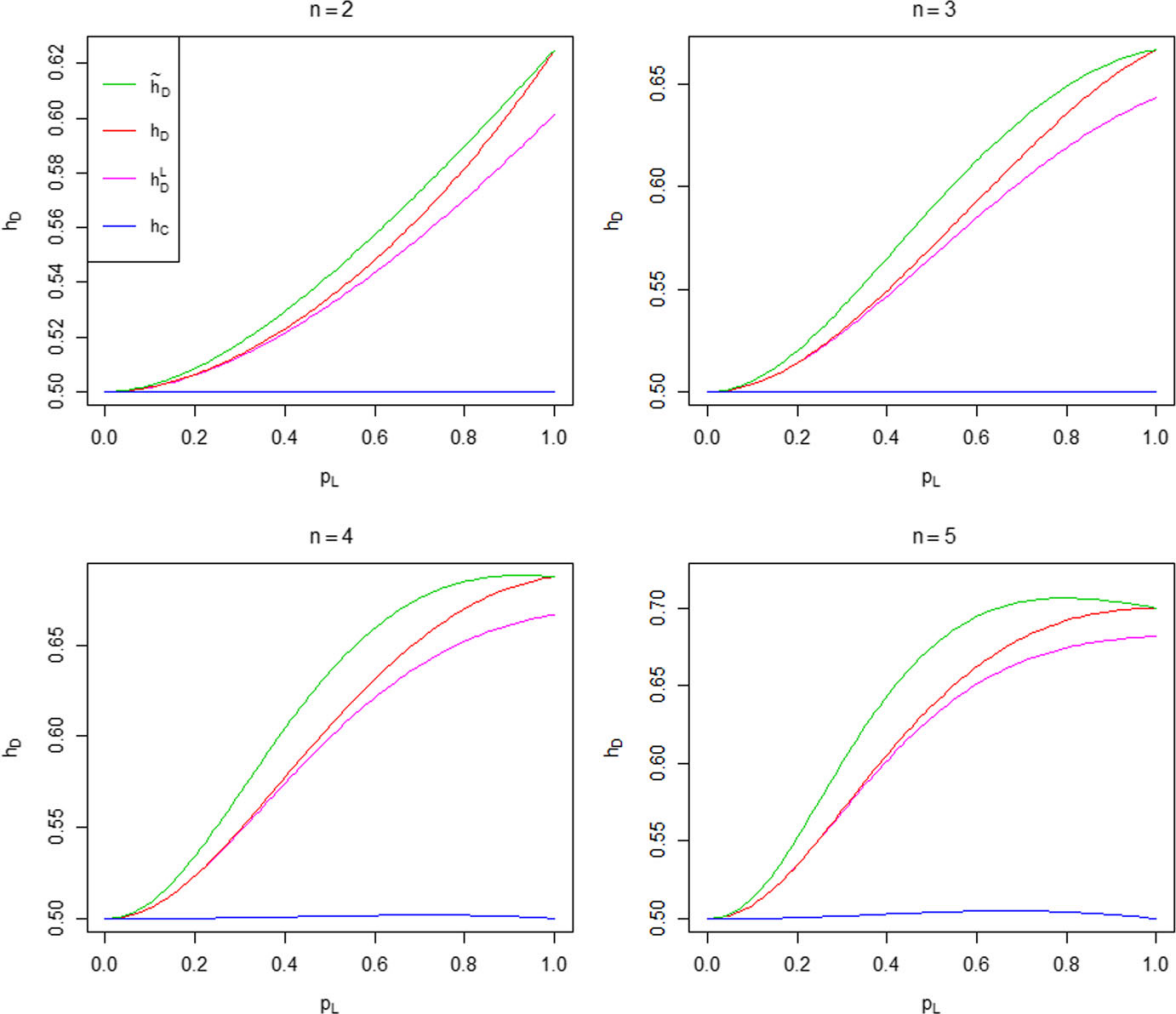
Herd immunity levels for fixed $R_0=2$ with common household size $n \in \{2,3,4,5\}$ and $\delta =\gamma =1$

In this subsection we assume that $\gamma =1$ and, where applicable, $\delta =1$ also. We begin by plotting in Fig. 6 how $h_D$, $h_D^L$, ${\tilde{h}}_D$ and $h_C$ vary with $p_L$ for a common household size *n* when $\lambda _G$ is chosen so that $R_0=2$ is fixed.

Figure 6 provides an illustration of Theorem 4.11 and support for its extension in Conjecture 4.12; we observe ${\tilde{h}}_D>h_C$ throughout Fig. 6. We also observe from Fig. 6 that we have $h_D>h_D^L$. Analytical comparison of $h_D^L$ and $h_D$ is often not tractable, however we can show that $h_D>h_D^L$ for $p_L$ sufficiently close to 1 as follows. When $p_L = 1$, under the SIR model disease-induced herd immunity leaves households either fully susceptible or fully non-susceptible. As noted in the discussion following Theorem 4.1, this corresponds to the worst possible vaccination strategy for a given coverage, implying $h_D>h_D^L$, since under the SEIR model there may be households in which only the initial case in that household is non-susceptible.

**Fig. 7:**
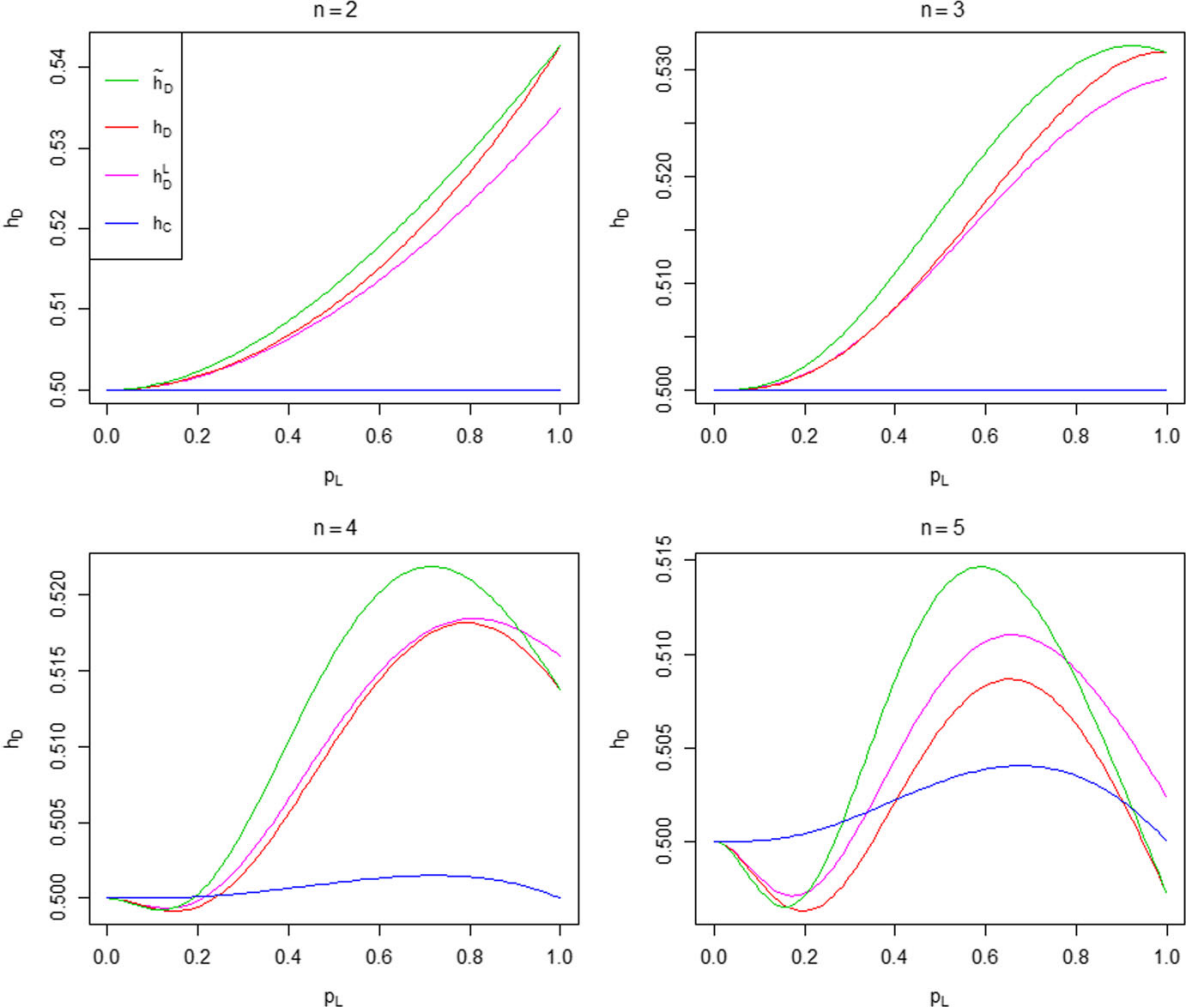
Herd immunity levels for fixed $R_0=2$ with household size distribution such that ${\tilde{\alpha }}_1={\tilde{\alpha }}_n=0.5$ for $n \in \{2,3,4,5\}$

In Fig. 7 we consider the same comparisons as in Fig. 6, but with a variable household size distribution. Specifically, we take ${\tilde{\alpha }}_1={\tilde{\alpha }}_n=0.5$ for some $n>1$; half the individuals in the population are in households of size 1 and the other half are in households of size *n*. This implies that $\alpha _1 = n/(n+1)$ and $\alpha _n = 1/(n+1)$.

A first observation based on Fig. 7 is that the behaviour near $p_L=0$ is consistent with the predictions of Sect. 4.3. Specifically, since $E[{\tilde{H}}] = (n-1)/2$ and $\textrm{var}({\tilde{H}})=(n-1)^2/4$, Theorem 4.8 predicts that $h_C>{\tilde{h}}_D$ near $p_L=0$ if and only if $n\ge 4$. There is contrasting behaviour in terms of the shape of the herd immunity levels as *n* increases. When $n=2$ and $n=3$, $h_C$ is the smallest of the considered herd immunity levels for all $p_L$. By contrast, when $n=4$ or $n=5$ there are values of $p_L$ such that $h_C$ is the largest of the considered herd immunity levels. As *n* increases in Fig. 7, the approximation ${\tilde{h}}_D$ for $h_D$ gets worse, cf. Sect. 3.2.3. Introducing a latent period does not necessarily lead to a reduction in the disease-induced herd immunity level; we observe that $h_D^L>h_D$ when $n=4$ and $n=5$.

Note that in Figs. 6 and 7 we have $h_C \ge 1-1/R_0$, with strict inequality unless all households are of size 3 or less; this follows from Theorem 1 of Ball et al. (2016).

### Real-world household size distributions

This section is motivated by the study in Britton et al. (2020), which considers the influence of population heterogeneity on the disease-induced herd immunity level for the COVID-19 pandemic. Britton et al. (2020) uses a Markov SEIR model in a population that is structured by age and activity level, in which for all individuals the exposed and infectious periods follow $\textrm{Exp}(1/3)$ and $\textrm{Exp}(1/4)$ distributions, respectively, with the unit of time being a day. Thus, the mean exposed and infectious periods are 3 and 4 days, respectively. Using the approximation to ${h}_D$ described in Sect. 1, Britton et al. (2020) find that, when $R_0=2.5$, $h_D$ for a homogeneously mixing model and $h_C$ are both $60\%$; for the model with both age and activity structure, $h_D$ is reduced to $43.0\%$.

The aim of the present numerical study is to investigate the effect of household structure on $h_D$, using a model with the above values of $\delta$ and $\gamma$ and a range of real-world household size distributions. In order to achieve that we need a way of calibrating models with different choices of $(\lambda _L, \lambda _G)$. One possibility is to keep the basic reproduction number $R_0$ fixed. However, $R_0$ is not uniquely defined for household models. The definition in Sect. 2.2 uses so-called rank generations and a different value for $R_0$ would typically be obtained if real-time generations were used instead, as for example in Neal and Theparod (2019). In practice, for an emerging epidemic, $R_0$ is often estimated indirectly, via an estimate of the epidemic’s early exponential growth rate *r*; see, for example, Wallinga and Lipsitch (2007). For the multitype SEIR model used in Britton et al. (2020), $R_0$ and *r* satisfy

5.1 $$\begin{aligned} R_0=\left( 1+\frac{r}{\delta }\right) \left( 1+\frac{r}{\gamma }\right) ; \end{aligned}$$

see Sections 1.3.1 and 1.5 of the supplementary material of Trapman et al. (2016). Note that the relationship (5.1) between $R_0$ and *r* is the same for all models in this class of multitype Markov SEIR epidemics and in particular matches that for the homogeneously mixing Markov SEIR model (Trapman et al. 2016).

We adopt the following method of calibrating models with different $(\lambda _L, \lambda _G)$, based on the early exponential growth rate *r*. For a given choice of $R_0$ in Britton et al.’s model, which we denote by $R_0^\textrm{BBT}$, we use (5.1) to calculate the corresponding value of the early exponential growth rate *r* under a multitype SEIR model. Then for a given value of $\lambda _L \in [0, \infty )$, we choose $\lambda _G$ so that the early exponential growth rate of our households SEIR model equals *r*; see Appendix C for details. As previously, we use the local infection probability $p_L = 1-\phi (\lambda _L)$ in the figures.

We consider real-world household size distributions from demographically diverse countries. Note that the exact distribution is not available for some countries we consider and hence is estimated by maximum likelihood estimation using the available data (mean household size and summaries of the household size distribution). The household structures, their corresponding sources and estimation procedures are given in Appendix D.

**Fig. 8:**
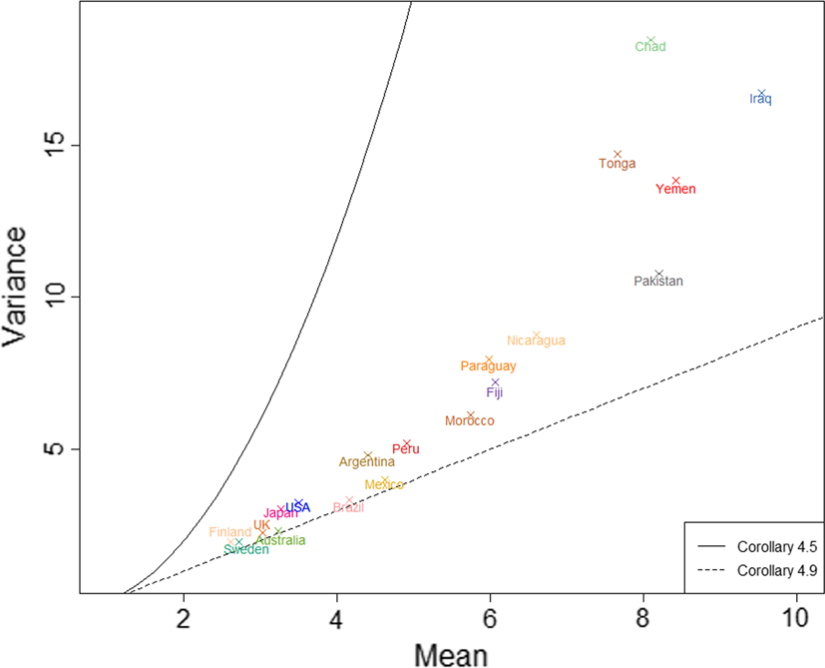
Critical values of $(\mu _{{\tilde{H}}},\sigma ^2_{{\tilde{H}}})$ from Corollaries 4.5 and 4.9, together with values of these quantities for several countries’ household size distributions

We begin by considering weakly locally infectious epidemics and just supercritical epidemics in Fig. 8, which illustrates Corollaries 4.5 and 4.9. This implicitly gives orderings of ${\tilde{h}}_D$ and $h_C$ for such epidemics, for a range of realistic household size distributions. Countries with $(\mu _{{\tilde{H}}},\sigma ^2_{{\tilde{H}}})$ below (above) the solid curve have ${\tilde{h}}_D>h_C$ (${\tilde{h}}_D<h_C$) for just supercritical epidemics in the highly locally infectious case; those below (above) the dashed curve have ${\tilde{h}}_D>h_C$ (${\tilde{h}}_D<h_C$) in the weakly locally infectious case.

The region enclosed between the solid and dashed black curves in Fig. 8 represents the set of values of $(\mu _{{\tilde{H}}},\sigma ^2_{{\tilde{H}}})$ for which Corollary 4.5 and Corollary 4.9 give different orderings of ${\tilde{h}}_D$ and $h_C$. We see that all countries considered have household size distributions in this set; though some are very close to the critical line $E[{\tilde{H}}-1]=\textrm{var}({\tilde{H}})$ in the weakly locally infectious case.

**Fig. 9:**
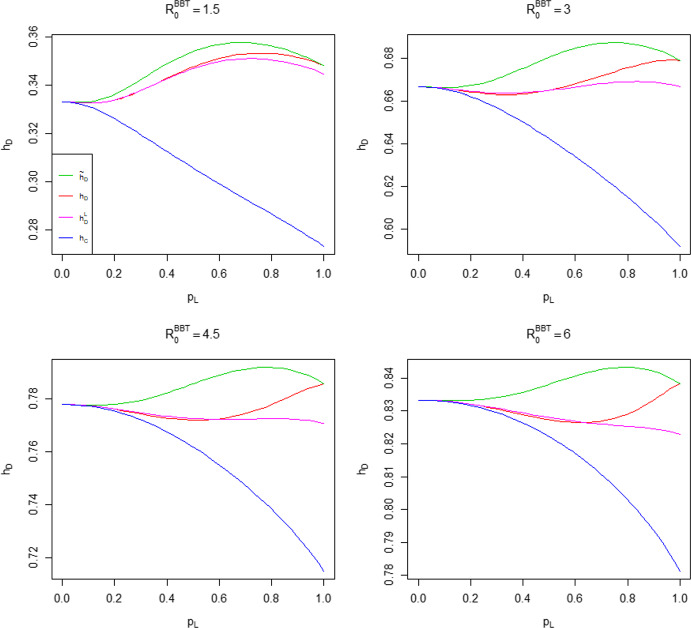
Herd immunity levels maintaining a fixed growth rate *r* implied by a given value of $R_0^\textrm{BBT}$, for the UK household size distribution

**Fig. 10:**
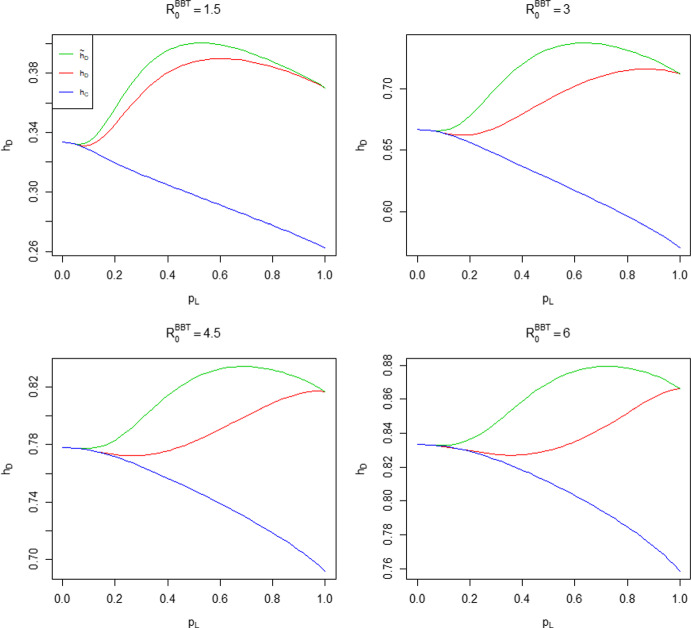
Herd immunity levels maintaining a fixed growth rate *r* implied by a given value of $R_0^\textrm{BBT}$, for Morocco’s household size distribution

We now explore the various herd immunity levels in our SIR and SEIR models, using the household size distributions of the UK (Fig. 9) and Morocco (Fig. 10) as exemplars. These countries are chosen because of their quite different household size distributions (cf. Fig. 8). The computation of $h_D^L$ is omitted for Morocco as its calculation becomes numerically infeasible, since the dimension of the system of ODEs (2.8) becomes too large owing to the high maximum household size.

Considering the UK household size distribution, which has $\mu _{{\tilde{H}}}=3.02$ and $\sigma ^2_{{\tilde{H}}}=2.26$, we see that $h_D>h_C$, which is as expected given we have observed $h_D<h_C$ only in cases where household sizes have very high variability. We also observe less variation in $h_D^L$ than in the other herd immunity levels. Increasing $R_0^\textrm{BBT}$ leads to the growth rate *r* being fixed at a higher value, in turn causing higher herd immunity levels. We also observe that $h_D^L$ and $h_D$ are very close for fixed *r* as $p_L$ increases from 0, until around $p_L=0.6$.

We observe very similar qualitative behaviour for other household size distributions, as shown for the Morocco household size distribution (which has $\mu _{{\tilde{H}}}=5.74$ and $\sigma ^2_{{\tilde{H}}}=6.12$) in Fig. 10.

**Fig. 11:**
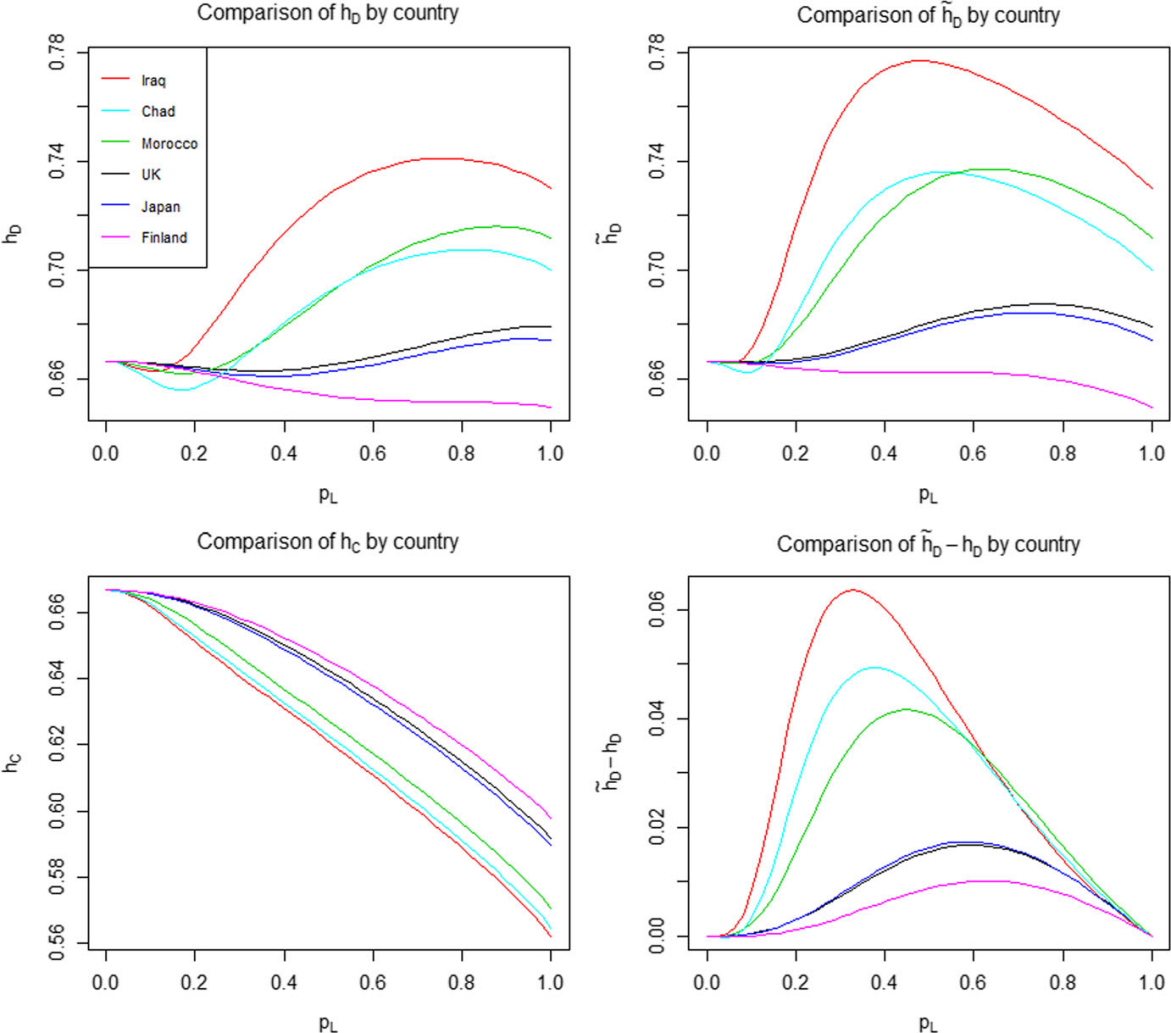
Comparison of $h_D$, ${\tilde{h}}_D$, ${\tilde{h}}_D-h_D$ and $h_C$ respectively by country for $R_0^\textrm{BBT}=3$ (with *r* held fixed) comparing Iraq, Chad, Morocco, UK, Japan and Finland

We now explore the quantitative differences between the herd immunity levels in detail for a wider range of countries’ household size distributions. Specifically, in Fig. 11 we compare the various herd immunity levels between several countries in the absence of a latent period, with $R_0^\textrm{BBT}=3$. (Estimates of $R_0$ for COVID-19 vary greatly even for the same country, but other choices for $R_0^\textrm{BBT}$ produce qualitatively similar results.)

We observe ${\tilde{h}}_D>h_D$ in Fig. 11, as well as ${\tilde{h}}_D=h_D$ at $p_L=0$ and $p_L=1$. For countries with generally smaller household sizes (i.e. Finland, Japan and the UK), ${\tilde{h}}_D$ and $h_D$ are very close in value. Countries with a larger value of $\mu _{{\tilde{H}}}$ give larger values for $h_D$ and ${\tilde{h}}_D$ but lower values of $h_C$. We generally observe ${\tilde{h}}_D>h_C$; the exceptions to this are for Morocco, Finland and Japan when $p_L$ is close to zero, and even then the difference between ${\tilde{h}}_D$ and $h_C$ is very small. We see $h_C$ decreases monotonically with $p_L$, whereas ${\tilde{h}}_D$ and $h_D$ are not monotone in their dependence on $p_L$. Finally, considering the last plot in Fig. 11, we find that the difference ${\tilde{h}}_D-h_D$ is maximised at a smaller value of $p_L$, with a larger maximum difference, when $\mu _{{\tilde{H}}}$ is larger.

## Concluding comments

We have presented a general framework for investigating disease-induced herd immunity in epidemic models with household structure. Calculating the disease-induced herd immunity level $h_D$ for such models is not straightforward and we have introduced a useful approximation ${\tilde{h}}_D$, which is more amenable to analysis. In sharp contrast to most forms of heterogeneous mixing, for which $h_D$ is *less* than the vaccine-induced herd immunity level $h_C$, the imposition of household structure generally leads to $h_D$ being *greater* than $h_C$, unless the variability in the household size distribution is sufficiently large. This is proved using ${\tilde{h}}_D$ for epidemics which are either highly or weakly locally infectious, and numerical studies support the conjecture that it holds more generally.

It would be worthwhile to consider more fully the impact of restrictions, such as lockdown, on ${\hat{h}}_D$, the disease-induced herd immunity level when restrictions are in place. In Sect. 3.2.2, we give an example where such restrictions affect only the global infection parameter $\lambda _G$, for which the impact of the restrictions on ${\hat{h}}_D$ is minimal; moreover, the approximation of ${\hat{h}}_D$ by ${\tilde{h}}_D$ improved with increasing restrictions. Similar results were found with other examples. However, in that example restrictions were applied uniformly with time which is unlikely to be the case in practice. Further, in practice restrictions may also affect the local infection rate $\lambda _L$; indeed it is not hard to envisage scenarios in which $\lambda _L$ might increase.

Another worthwhile avenue for future research is to consider models which combine household structure with other forms of heterogeneous mixing. This can be done using the multitype households model and a similar approximation to ${\tilde{h}}_D$ for the disease-induced herd immunity level can be calculated using results in Ball and Lyne (2001). We are currently investigating this for a model with activity levels, as in Britton et al. (2020), and also household structure.

We have assumed throughout that the individual-to-individual local infection rate $\lambda _L$ is independent of household size *n*. Although this assumption is often made with household models and is usually reasonable for small *n*, such as in the UK, Sweden and Finland household size distributions, it is less easily justified for countries with large household sizes, such as Iraq, Pakistan and Chad. One would expect $\lambda _L$ to decrease with *n* and it would be interesting to explore the consequent impact on $h_D$. Note that the results of Sect. 4.2 concerning the highly locally infectious case are unaffected but other results may change.

Throughout a large part of this work we have used ${\tilde{h}}_D$ as an approximation to $h_D$. The only models in which we have computed $h_D$ are those in which the infectious and latent periods follow exponential distributions. In real-life epidemics, the distributions of these quantities are usually far from exponential. Moreover, the impact of departures from exponential distributions on epidemic properties is usually greater in models incorporating small mixing groups, such as households. Although it is possible in principle to use the method of stages to extend the deterministic model in Sect. 2.4 to include Erlang distributed infectious and latent periods, and to allow for varied local and global infection rates between stages of infection, in practice, the number of ODEs soon becomes infeasible. However, it is straightforward to calculate ${\tilde{h}}_D$ for such models, and indeed for models in which individuals have infectivity profiles (for example, Goldstein et al. (2009)), since such calculation only requires final outcome properties of an epidemic. We have found that ${\tilde{h}}_D>h_D$ in most of our numerical studies with exponentially distributed infectious and latent periods, and that the difference is typically small unless the mean household size is large. We expect a similar conclusion to hold for models with other, more realistic, choices of infectious and latent period distributions.

## Figures and Tables

**Fig. 12:**
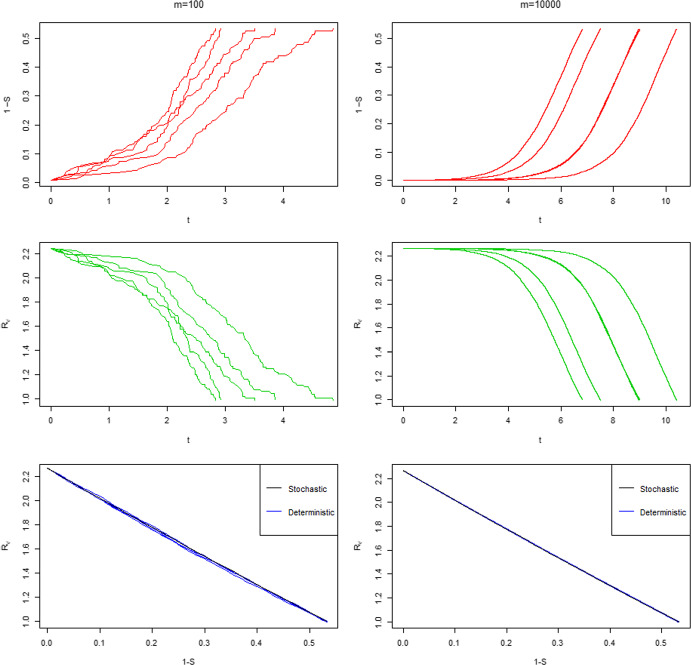
Realised trajectories of $R_V^{(m)}(t)$ and $1-S^{(m)}(t)$ for $m=100$ (left column) and $m=10000$ (right column), with $(\lambda _G,\lambda _L,\gamma )=(2,0.25,1)$ and an equal number of households of size 1 and size 2 with a single initial infective residing in a household of size 2. The top row plots the trajectories of $1-S^{(m)}(t)$ against *t*. The second row plots the trajectories of $R_V^{(m)}(t)$ against *t*. The final row plots the trajectories of $R_V^{(m)}(t)$ against $1-S^{(m)}(t)$, with the epidemic progressing from left to right and with the corresponding deterministic trajectory superimposed

## Data Availability

This manuscript has no associated data.

## A Convergence of $H_D^{(m)}$ to $h_D$

In this appendix, we comment briefly on a possible approach to proving the conjecture in Sect. 3.2.1 that $H_D^{(m)}{\mathop {\longrightarrow }\limits ^{\textrm{p}}}h_D^L$ as $m\rightarrow \infty$. We also present some numerical evidence in support of that conjecture for the SIR model and for the conjecture underlying the calculation of $h_D$ in that model.

A possible proof of $H_D^{(m)}{\mathop {\longrightarrow }\limits ^{\textrm{p}}}h_D^L$ as $m\rightarrow \infty$ is to extend the theory of Barbour and Reinert (2013) to the households SEIR model. Briefly, such an extension would imply that, as $m \rightarrow \infty$, in the event of a major outbreak, the process $\left\{ m^{-1}\varvec{H}^{(m)}(t):t \ge 0\right\}$, where $\varvec{H}^{(m)}(t)=\left( H^{(m)}_{s,e,i,r}(t): (s,e,i,r) \in {\mathscr {H}}^{(n_{\max })}\right)$ (see Sect. 2.4), converges in probability to a random time translation of a deterministic process $\{{\tilde{\varvec{h}}}(t):-\infty<t <\infty \}$ in which the fraction of the population that is either exposed or infective converges to 0 as $t \rightarrow -\infty$. The random time translation arises from the randomness in the initial behaviour of the approximating branching process (see Sect. 2.2) before it settles into its exponentially growing regime. One can use ${\tilde{\varvec{h}}}(t)$ to define (deterministic) analogues of $R_V^{(m)}(t)$ and $S^{(m)}(t)$ above, ${\tilde{R}}_V(t)$ and ${\tilde{s}}(t)$ say, and $h_D^L=1-{\tilde{s}}({\tilde{t}}_*)$, where ${\tilde{t}}_*=\inf \{t \in (-\infty , \infty ): {\tilde{R}}_V(t) \le 1\}$.

In Table 2 we present an example showing empirical evidence in support of this conjecture for the households SIR model when $T_E \sim \textrm{Exp}(\gamma )$. We also see in the bottom row of Table 2 evidence that $m \textrm{var}(H_D^{(m)})$ converges to a constant as $m\rightarrow \infty$, which is consistent with a central limit theorem for $H_D^{(m)}$.

**Table 2:** Empirical evidence for the convergence of $H_D^{(m)}$ to a limiting value as $m \rightarrow \infty$, using 10000 simulations of major outbreaks with $(\lambda _G,\lambda _L,\gamma )=(2,0.25,1)$ and an equal number of households of size 1 and size 2

*m*	${10}^2$	${10}^3$	${10}^4$	${10}^5$
${\bar{H}}_D^{(m)}$	0.533495	0.532459	0.532260	0.532240
${\hat{\sigma }}^{(m)}$	$1.70\times 10^{-2}$	$1.67\times 10^{-3}$	$5.29 \times 10^{-4}$	$1.67 \times {10}^{-4}$
$m({\hat{\sigma }}^{(m)})^2$	$2.90 \times 10^{-2}$	$2.78 \times 10 ^{-3}$	$2.80 \times 10^{-3}$	$2.80 \times 10^{-3}$

The sample mean $({\bar{H}}_D^{(m)})$ and sample standard deviation (${\hat{\sigma }}^{(m)}$) of $H_D^{(m)}$ are given for various total number of households (*m*). The mean appears to converge toward a fixed value, with the standard deviation appearing to decrease toward 0 as $m \rightarrow \infty$

Recall from Sect. 3.2.1 that we calculate $h_D^L$ by considering a sequence of deterministic epidemics in which the initial fraction of the population that is infected tends to 0. Further, we conjecture that $h_D^L(\varvec{\epsilon }^{(k)})\rightarrow h_D^L$ as $k \rightarrow \infty$ for any sequence $(\varvec{\epsilon }^{(k)})$ satisfying $\sum _{(s,e,i,r) \in {\mathscr {H}}^{(n_{\max })}_+}\epsilon _{s,e,i,r}^{(k)} \downarrow 0$ and $\sum _{(s,e,i,r) \in {\mathscr {H}}_{0\text {rec}}} \epsilon _{s,e,i,r}^{(k)} \uparrow 1$ as $k \rightarrow \infty$. We give an example for an SIR model in Table 3.

**Table 3:** Empirical evidence of convergence of $h_D(\epsilon )$ to $h_D$, using $(\lambda _G,\lambda _L,\gamma )=(2,0.25,1)$ and an equal number of households of size 1 and size 2, with an initial fraction $\epsilon$ of infected households, where $\epsilon =\sum _{(s,e,i,r) \in {\mathscr {H}}^{(n_{\max })}_+} \epsilon _{s,e,i,r}$

	$\epsilon$	$10^{-2}$	$10^{-3}$	$10^{-4}$	$10^{-5}$
Initially	Size 1	0.532274	0.532242	0.532238	0.532239
infected	Size 2	0.532032	0.532218	0.532237	0.532239
households	Sizes 1 & 2	0.532312	0.532246	0.532237	0.532239

The different rows correspond to different choices of initial condition in terms of which household sizes contain initial infectives. In the first row, all initial infectives reside in size 1 households, and in the second row all initial infectives reside in size 2 households. In the third row, a fraction $\frac{\epsilon }{2}$ households contain one initial infective and a fraction $\frac{\epsilon }{2}$ households contain two initial infectives. As $\epsilon \downarrow 0$, $h_D(\epsilon )$ approaches a value consistent with Table 2. Smaller values of $\epsilon$ give the same value as $h_D(10^{-5})$ to this level of accuracy

Tables 2 and 3 suggest that $\lim _{m\rightarrow \infty }H_D^{(m)}$ can be computed as the solution of the appropriate corresponding deterministic equations, along with the stopping condition $R_V(t)=1$.

Figure 12 gives an example showing that, as *m* increases, the trajectories $\{S^{(m)}(t)\}$ and $\{R_V^{(m)}(t)\}$ become smoother, with a random time translation capturing the time it takes for the stochastic epidemic to take off. As a result, the trajectories of $R_V^{(m)}(t)$ and $1-S^{(m)}(t)$ against *t* can, for large *m*, be thought of as random time shifts of the corresponding deterministic trajectories. The random time shift is absent, however, when considering $R_V^{(m)}(t)$ against $1-S^{(m)}(t)$, since the time shifts of $R_V^{(m)}(t)$ and $1-S^{(m)}(t)$ are the same for a given realisation of the process. In light of the definition above of $H_D^{(m)}$, we thus find, for large *m*, that $H_D^{(m)}\simeq \lim _{\varvec{\epsilon } \rightarrow {\varvec{0}}}h_D(\varvec{\epsilon })$. Owing to the random time shift, however, it is not the case that $T_*^{(m)}\simeq t_*$ even for large *m*.

## B Proofs

### B.1 Proof of Theorem 4.7

#### Proof

Let $\pi \in (0,1)$. Using (4.5), $R_{DI}(\pi )=\lambda _G\textrm{E}[T_I][(1-p)\pi +np\pi ^n]$. Note that the proportion *z* infected in the first epidemic satisfies $z=1-(1-p)\pi -p\pi ^n$. We compare $R_{DI}(\pi )$ with the corresponding reproduction number $R_U(\pi )$, when this fraction *z* of the population is vaccinated uniformly at random. Using (4.1) (cf. (4.2)),

$$\begin{aligned} R_U(\pi )&=\lambda _G\textrm{E}[T_I][1-z+p(n-1)(1-z)^2]\\&=\lambda _G\textrm{E}[T_I]\left\{ (1-p)\pi +p\pi ^n+p(n-1)[(1-p)\pi +p\pi ^n]^2\right\} . \end{aligned}$$

Hence,

$$\begin{aligned} R_U(\pi )-R_{DI}(\pi )=p(n-1)\lambda _G\textrm{E}[T_I]\left( \left[ (1-p)\pi +p\pi ^n\right] ^2 -\pi ^n\right) . \end{aligned}$$

Let $h_n(\pi )=\pi ^{\frac{n}{2}-1}-p\pi ^{n-1}$. Elementary algebra shows that $R_U(\pi )-R_{DI}(\pi )<0$ if $h_n(\pi )>1-p$, $R_U(\pi )-R_{DI}(\pi )=0$ if $h_n(\pi )=1-p$, and $R_U(\pi )-R_{DI}(\pi )>0$ if $h_n(\pi )<1-p$. Now $h_2(\pi )=1-p\pi >1-p$ for all $\pi \in (0,1)$, so when $n=2$, $R_U(\pi )<R_{DI}(\pi )$ for all $\pi \in (0,1)$, whence ${\tilde{h}}_D>h_C$ for all $\lambda _G$ (more precisely all $\lambda _G$ such that the epidemic is supercritical). Suppose $n \ge 3$. Now $h_n(0)=0$, $h_n(1)=1-p$ and

$$\begin{aligned} h_n^{\prime }(\pi )=\left( \frac{n}{2}-1\right) \pi ^{\frac{n}{2}-2}-p(n-1)\pi ^{n-2}. \end{aligned}$$

If $p \le \frac{n-2}{2(n-1)}$ then $h_n^{\prime }(\pi )> 0$ for all $\pi \in (0,1)$, so $h_n(\pi )<1-p$ for all $\pi \in (0,1)$, leading to ${\tilde{h}}_D<h_C$ for all $\lambda _G$. Suppose that $p > \frac{n-2}{2(n-1)}$ and let $\pi _n^{*}(p)=\left( \frac{n-2}{2p(n-1)} \right) ^{\frac{2}{n}}$. Then $h_n^{\prime }(\pi )>0$ for $\pi \in (0, \pi _n^{*}(p))$ and $h_n^{\prime }(\pi )<0$ for $\pi \in (\pi _n^{*}(p),1)$. Hence, $h_n(\pi )=1-p$ has a unique solution, ${\hat{\pi }}_n(p)$ say, in (0, 1). Further, $h_n(\pi )<1-p$ for $\pi \in (0, {\hat{\pi }}_n(p))$, implying $R_U(\pi )>R_{DI}(\pi )$, and $h_n(\pi )>1-p$ for $\pi \in ({\hat{\pi }}_n(p),1)$, implying $R_U(\pi )<R_{DI}(\pi )$. It follows that there exists $\lambda _G^{*}(n,p)$ such that ${\tilde{h}}_D>h_C$ if $\lambda _G < \lambda _G^{*}(n, p)$, and ${\tilde{h}}_D<h_C$ if $\lambda _G > \lambda _G^{*}(n,p)$. The change in behaviour occurs when $R_{DI}({\hat{\pi }}_n(p))=R_U({\hat{\pi }}_n(p))=1$. Substituting ${\hat{\pi }}_n(p)$ into the above expression for $R_{DI}(\pi )$ yields $\lambda _G^{*}(n, p)=\left[ \textrm{E}[T_I]{\hat{\pi }}_n(p)\left( 1-p+np{{\hat{\pi }}_n(p)}^{n-1}\right) \right] ^{-1}$, as required. $\square$

### B.2 Proof of Theorem 4.8

For $k=0,1,\dots ,n$, let $n_{[k]}=n!/(n-k)!$ denote the falling factorial. Consider the single-household epidemic ${\tilde{E}}_n(\lambda _L,\pi )$ described in Sect. 2.3. Let ${\tilde{S}}_n=n-{\tilde{T}}_n$ be the number of susceptibles remaining at the end of the epidemic. Define $\mu _n(\lambda _L)$ as in Sect. 2.2 and also define, for $k=1,2$,

$$\begin{aligned} {\hat{\mu }}_{n,k}(\lambda _L,\pi )=\textrm{E}[({\tilde{S}}_n)_{[k]}]. \end{aligned}$$

Recall we assume that $\textrm{E}[T_I]=1$ and $\textrm{var}(T_I)<\infty$. We start with a preliminary lemma.

#### Lemma B.1

For $n=1,2,\dots$, we have $\mu _n(0)=1$ and $\mu _n^{\prime }(0)=n-1$. Let $\pi =\pi (\lambda _L)$. For $n=1,2,\dots$ and $k=1,2$, we have ${\hat{\mu }}_{n,k}(0,\pi (0))=n_{[k]}\pi (0)^k$, $\frac{\partial }{\partial \lambda _L}{\hat{\mu }}_{n,k}(0,\pi (0))=-kn_{[k+1]}\pi (0)^k(1-\pi (0))$ and $\frac{\partial }{\partial \pi }{\hat{\mu }}_{n,k}(0,\pi (0))=n_{[k]}k\pi (0)^{k-1}$. Further, $\frac{\partial ^2}{\partial \pi ^2}{\hat{\mu }}_{n,1}(0,\pi (0))=0$ and $\frac{\partial ^2}{\partial \lambda _L\partial \pi }{\hat{\mu }}_{n,1}(0,\pi (0))=-n(n-1)(1-2\pi (0))$, where all derivatives are evaluated at $\lambda _L=0$. For example, $\frac{\partial }{\partial \lambda _L}{\hat{\mu }}_{n,k}(0,\pi (0))=\frac{\partial }{\partial \lambda _L}{\hat{\mu }}_{n,k}(\lambda _L,\pi )\bigg |_{(\lambda _L,\pi )=(0,\pi (0))}$.

#### Proof

We make use of Gontcharoff polynomials; see Lefèvre and Picard (1990), Sect. 2, for details. For a given sequence $U=u_0,u_1,\dots$ of real numbers, the corresponding Gontcharoff polynomials, $G_0(x\mid U), G_1(x\mid U), \dots$, are defined by

B.1 $$\begin{aligned} \sum _{i=0}^nn_{[i]}u_i^{n-i}G_i(x\mid U)=x^n\;\;\;\;\;(n=0,1,\dots ). \end{aligned}$$

We consider the real sequence with $u_i=\phi (i\lambda _L)$. For $k=1,2$ and $i=0,1,\dots$, let $G_{i,k}(\lambda _L)=G_i(1\mid E^kU)$, where $E^kU$ denotes the sequence $u_k,u_{k+1},\dots$. Using Ball (2019), Proposition 3.1 and Properties 2.1 and 2.2, gives

B.2 $$\begin{aligned} \mu _n(\lambda _L)=n-\sum _{i=1}^{n-1} (n-1)_{[i]}\phi (i\lambda _L)^{n-i}G_{i-1,1}(\lambda _L) \end{aligned}$$

and

B.3 $$\begin{aligned} {\hat{\mu }}_{n,k}(\lambda _L,\pi )=\sum _{i=k}^nn_{[i]}\phi (i\lambda _L)^{n-i}\pi ^iG_{i-k,k}(\lambda _L)\;\;\;\;\; (k=1,2). \end{aligned}$$

Consider $G_{i,k}(0)$ for $k=1,2$. Substituting $\lambda _L=0$ into (B.1) implies that $\sum _{i=0}^nn_{[i]}G_{i,k}(0)=1$. Recalling $n_{[0]}=1$ then gives $G_{0,k}(0)=1$, leading to $G_{i,k}(0)=\delta _{i,0}$ for $i=0,1,\dots$, where

$$\begin{aligned} \delta _{i,j} = {\left\{ \begin{array}{ll} 1 &{} \hbox { if}\ i=j\\ 0 &{} \hbox { if}\ i\ne j. \end{array}\right. } \end{aligned}$$

Differentiating (B.1) with respect to $\lambda _L$ and setting $\lambda _L=0$ yields

$$\begin{aligned} \sum _{i=0}^nn_{[i]}\left( G_{i,k}^{\prime }(0)-G_{i,k}(0)(n-i)(i+k)\right) =0. \end{aligned}$$

Using $G_{i,k}(0)=\delta _{i,0}$ then gives $G_{i,k}^{\prime }(0)=k\delta _{i,1}$, for $i=0,1,\dots$ and $k=1,2$. We now take the appropriate partial derivatives of (B.2), (B.3) and substitute $\lambda _L=0$, noting that $\phi (0)=1$ and $\phi ^{\prime }(0)= - \textrm{E}[T_I] =-1$. Differentiating (B.2) with respect to $\lambda _L$ gives

B.4 $$\begin{aligned} \begin{aligned} \mu _n^{\prime }(\lambda _L)=-&\sum _{i=1}^{n-1}(n-1)_{[i]}(n-i)i\phi (i\lambda _L)^{n-i-1}\phi ^{\prime }(i\lambda _L)G_{i-1,1}(\lambda _L)\\ -&\sum _{i=1}^{n-1}(n-1)_{[i]}\phi (i\lambda _L)^{n-i} G^{\prime }_{i-1,1}(\lambda _L). \end{aligned} \end{aligned}$$

Substituting $\lambda _L=0$ into (B.2), (B.4) and (B.3) respectively then establishes that $\mu _n(0)=1$, $\mu _n^{\prime }(0)=n-1$ and ${\hat{\mu }}_{n,k}(0,\pi (0))=n_{[k]}\pi (0)^k$.

Differentiating (B.3) with respect to $\lambda _L$, we find

B.5 $$\begin{aligned} \begin{aligned} \frac{\partial }{\partial \lambda _L}{\hat{\mu }}_{n,k}(\lambda _L,\pi )=&\sum _{i=k}^nn_{[i]}(n-i)i\phi (i\lambda _L)^{n-i-1}\phi ^{\prime }(i\lambda _L)\pi ^iG_{i-k,k}(\lambda _L)\\ +&\sum _{i=k}^nn_{[i]}\phi (i\lambda _L)^{n-i}\pi ^iG^{\prime }_{i-k,k}(\lambda _L), \end{aligned} \end{aligned}$$

from which letting $\lambda _L=0$ gives $\frac{\partial }{\partial \lambda _L}{\hat{\mu }}_{n,k}(0,\pi (0))=-kn_{[k+1]}\pi (0)^k(1-\pi (0))$. Next, taking the derivative of (B.3) with respect to $\pi$ yields

B.6 $$\begin{aligned} \frac{\partial }{\partial \pi }{\hat{\mu }}_{n,k}(\lambda _L,\pi )=\sum _{i=k}^nn_{[i]}\phi (i\lambda _L)^{n-i}i\pi ^{i-1}G_{i-k,k}(\lambda _L), \end{aligned}$$

which gives $\frac{\partial }{\partial \pi }{\hat{\mu }}_{n,k}(0,\pi (0))=n_{[k]}k\pi (0)^{k-1}$ by setting $\lambda _L=0$. For the second derivatives, we require further differentiation. Firstly, differentiating (B.5) with respect to $\pi$, we have

B.7 $$\begin{aligned} \begin{aligned} \frac{\partial ^2}{\partial \lambda _L\partial \pi }{\hat{\mu }}_{n,k}(\lambda _L,\pi )=&\sum _{i=k}^nn_{[i]}(n-i)i^2\phi (i\lambda _L)^{n-i-1}\phi ^{\prime }(i\lambda _L)\pi ^{i-1}G_{i-k,k}(\lambda _L)\\ +&\sum _{i=k}^nn_{[i]}\phi (i\lambda _L)^{n-i}i\pi ^{i-1}G^{\prime }_{i-k,k}(\lambda _L). \end{aligned} \end{aligned}$$

Taking $\lambda _L=0$ and $k=1$ in (B.7), we find $\frac{\partial ^2}{\partial \lambda _L\partial \pi }{\hat{\mu }}_{n,1}(0,\pi (0))=-n(n-1)(1-2\pi (0))$. Finally, differentiating (B.6) with respect to $\pi$ gives

B.8 $$\begin{aligned} \frac{\partial ^2}{\partial \pi ^2}{\hat{\mu }}_{n,k}(\lambda _L,\pi )=\sum _{i=k}^nn_{[i]}\phi (i\lambda _L)^{n-i}i(i-1)\pi ^{i-2}G_{i-k,k}(\lambda _L). \end{aligned}$$

Letting $\lambda _L=0$ and $k=1$ in (B.8) causes all terms to vanish, so that $\frac{\partial ^2}{\partial \pi ^2}{\hat{\mu }}_{n,1}(0,\pi (0))=0$, which then establishes the final result of Lemma B.1. $\square$

We are now in a position to prove Theorem 4.8. For ease of exposition in the following proof, we denote $h_C(\lambda _L)$ by $c(\lambda _L)$. Similarly, we denote $h_D(\lambda _L)$ by $z(\lambda _L)$. Recall that $n_{\max }<\infty$, so all sums in the proof contain only finitely many terms and hence are easily differentiated.

#### Proof of Theorem 4.8

Suppose a fraction $c(\lambda _L)$ are vaccinated prior to an epidemic, such that the epidemic becomes critical. Note $c(0)=1-\frac{1}{\lambda _G}$ (recall $\textrm{E}[T_I] =1$). Now considering disease-induced herd immunity, assume that a fraction $z(\lambda _L)$ are infected in a first epidemic such that the second epidemic is critical. Let $\pi (\lambda _L)$ be the proportion who avoid global infection in the first epidemic. Then $z(0)=1-\frac{1}{\lambda _G}$ and $\pi (0)=\frac{1}{\lambda _G}$. We show that $c^{\prime }(0)=z^{\prime }(0)=\frac{\mu _{{\tilde{H}}-1}}{\lambda _G^2}$ and that

$$\begin{aligned} z^{\prime \prime }(0)-c^{\prime \prime }(0)=4\pi (0)^2(1-\pi (0))\left( \textrm{E}[{\tilde{H}}-1]-\textrm{var}({\tilde{H}})\right) , \end{aligned}$$

from which Theorem 4.8 follows immediately by Taylor’s theorem.

We begin by considering the derivatives of $c(\lambda _L)$ at $\lambda _L=0$. The post-vaccination threshold parameter ${\hat{R}}_U(c)$ defined at (3.1) satisfies ${\hat{R}}_U(c(\lambda _L))=1$, so by substituting $i=n-v$ in the inner sum in (3.1) we have that

B.9 $$\begin{aligned} \lambda _G\sum _{n=1}^{\infty }{\tilde{\alpha }}_n\sum _{i=1}^n\frac{i}{n}\left( {\begin{array}{c}n\\ i\end{array}}\right) (1-c(\lambda _L))^ic(\lambda _L)^{n-i}\mu _i({\lambda _L})=1. \end{aligned}$$

Let $$q_{n,i}(c)=\frac{i}{n}\left( {\begin{array}{c}n\\ i\end{array}}\right) (1-c)^ic^{n-i}$$. Then $\sum _{i=1}^nq_{n,i}(c)=1-c$, $\sum _{i=1}^nq^{\prime }_{n,i}(c)=-1$ and $\sum _{i=1}^nq^{\prime \prime }_{n,i}(c)=0$, by exchanging the order of derivative and summation. We also have $\sum _{i=1}^n(i-1)q_{n,i}(c)=(n-1)(1-c)^2$, so $\sum _{i=1}^n(i-1)q_{n,i}^{\prime }(c)=-2(n-1)(1-c)$. Differentiating (B.9) gives

B.10 $$\begin{aligned} \sum _{n=1}^{\infty }{\tilde{\alpha }}_n\sum _{i=1}^n\left[ q_{n,i}^{\prime }(c(\lambda _L))c^{\prime }(\lambda _L)\mu _i(\lambda _L)+q_{n,i}(c(\lambda _L))\mu _i^{\prime }(\lambda _L)\right] =0. \end{aligned}$$

Substituting $\lambda _L=0$ in (B.10) and recalling $c(0)=1-\frac{1}{\lambda _G}$ yields $c^{\prime }(0)=\frac{\mu _{{\tilde{H}}-1}}{\lambda _G^2}$. Differentiating (B.10) gives

B.11 $$\begin{aligned} \begin{aligned}&\sum _{n=1}^{\infty }{\tilde{\alpha }}_n\sum _{i=1}^n\left[ q_{n,i}^{\prime \prime }(c(\lambda _L))c^{\prime }(\lambda _L)^2+q^{\prime }_{n,i}(c(\lambda _L))c^{\prime \prime }(\lambda _L) \right] \mu _i(\lambda _L)\\&\qquad \qquad +\sum _{n=1}^{\infty }{\tilde{\alpha }}_n\sum _{i=1}^n2q_{n,i}^{\prime }(c(\lambda _L))c^{\prime }(\lambda _L)\mu _i^{\prime }(\lambda _L)\\&\qquad \qquad +\sum _{n=1}^{\infty }{\tilde{\alpha }}_n\sum _{i=1}^nq_{n,i}(c(\lambda _L))\mu _i^{\prime \prime }(\lambda _L)=0. \end{aligned} \end{aligned}$$

Let $A_n=\sum _{i=1}^nq_{n,i}(c(0))\mu _i^{\prime \prime }(0)$. Then we set $\lambda _L=0$ in (B.11) which yields

B.12 $$\begin{aligned} c^{\prime \prime }(0)=\sum _{n=1}^{\infty }{\tilde{\alpha }}_nA_n-4c^{\prime }(0)(1-c(0))\mu _{{\tilde{H}}-1}. \end{aligned}$$

Turning now to $z(\lambda _L)$, let ${\tilde{P}}_{n,i}(\lambda _L,\pi (\lambda _L))=\textrm{P}({\tilde{S}}_n=i)$ be the probability *i* members of a household avoid infection in the epidemic ${\tilde{E}}_n(\lambda _L,\pi (\lambda _L))$. Suppose all individuals infected (no longer susceptible) in the first epidemic are immune to infection in the second epidemic. Suppose the second epidemic is critical, so that $R_{DI}(\pi (\lambda _L))=1$. Then considering the remaining susceptibles yields

B.13 $$\begin{aligned} \lambda _G\sum _{n=1}^{\infty }{\tilde{\alpha }}_n\sum _{i=1}^n\frac{i}{n}{\tilde{P}}_{n,i}(\lambda _L,\pi (\lambda _L))\mu _i(\lambda _L)=1. \end{aligned}$$

Further, the proportion of the population infected in the first epidemic is given by (cf. (2.4))

B.14 $$\begin{aligned} z(\lambda _L)=1-\sum _{n=1}^{\infty }{\tilde{\alpha }}_n\frac{1}{n}{\hat{\mu }}_{n,1}(\lambda _L,\pi (\lambda _L)). \end{aligned}$$

Differentiating (B.13) gives

B.15 $$\begin{aligned}&\sum _{n=1}^{\infty }{\tilde{\alpha }}_n\sum _{i=1}^n\frac{i}{n}\left[ \mu _i(\lambda _L)\left\{ \frac{\partial }{\partial \lambda _L}\tilde{P}_{n,i}(\lambda _L,\pi (\lambda _L))+ \frac{\partial }{\partial \pi }\tilde{P}_{n,i}(\lambda _L,\pi (\lambda _L))\pi ^{\prime }(\lambda _L)\right\} \right] \nonumber \\&\qquad \qquad + \sum _{n=1}^{\infty }{\tilde{\alpha }}_n\sum _{i=1}^n\frac{i}{n}\left[ \mu _i^{\prime }(\lambda _L){\tilde{P}}_{n,i}(\lambda _L,\pi (\lambda _L))\right] =0, \end{aligned}$$

which can be used to solve for $\pi ^{\prime }(0)$ by setting $\lambda _L=0$. Applying Lemma B.1 and noting that ${\hat{\mu }}_{n,k}(\lambda _L,\pi (\lambda _L))=\sum _{i=1}^ni_{[k]}{\tilde{P}}_{n,i}(\lambda _L,\pi (\lambda _L))$ yields $\pi ^{\prime }(0)=(\pi (0)-2\pi (0)^2)\mu _{{\tilde{H}}-1}$. Differentiating (B.14), we have

B.16 $$\begin{aligned} z^{\prime }(\lambda _L)=-\sum _{n=1}^{\infty }{\tilde{\alpha }}_n\frac{1}{n}\left\{ \frac{\partial }{\partial \lambda _L}{\hat{\mu }}_{n,1}(\lambda _L,\pi (\lambda _L))+\frac{\partial }{\partial \pi }{\hat{\mu }}_{n,1}(\lambda _L,\pi (\lambda _L))\pi ^{\prime }(\lambda _L) \right\} .\nonumber \\ \end{aligned}$$

Substituting $\lambda _L=0$ in (B.16), we find that $z^{\prime }(0)=\frac{\mu _{{\tilde{H}}-1}}{\lambda _G^2}=c^{\prime }(0)$. Before proceeding with further differentiation, let $B_n=\frac{1}{n}\frac{\partial ^2}{\partial \lambda _L^2}{\hat{\mu }}_{n,1}(0,\pi (0))$. Differentiating (B.15), we reach

$$\begin{aligned} \begin{aligned}&\sum _{n=1}^{\infty }{\tilde{\alpha }}_n\sum _{i=1}^n\frac{i}{n}\left[ 2\mu _i^{\prime }(\lambda _L)\left\{ \frac{\partial }{\partial \lambda _L}\tilde{P}_{n,i}(\lambda _L,\pi (\lambda _L))+ \frac{\partial }{\partial \pi }\tilde{P}_{n,i}(\lambda _L,\pi (\lambda _L))\pi ^{\prime }(\lambda _L)\right\} \ \right] \\&\qquad +\sum _{n=1}^{\infty }{\tilde{\alpha }}_n\sum _{i=1}^n\frac{i}{n}\left[ \mu _i(\lambda _L)\left\{ \frac{\partial ^2}{\partial \lambda _L^2}\tilde{P}_{n,i}(\lambda _L,\pi (\lambda _L))+2\pi ^{\prime }(\lambda _L)\frac{\partial ^2}{\partial \lambda _L\partial \pi }\tilde{P}_{n,i}(\lambda _L,\pi (\lambda _L)) \right\} \right] \\&\qquad +\sum _{n=1}^{\infty }{\tilde{\alpha }}_n\sum _{i=1}^n\frac{i}{n}\left[ \mu _i(\lambda _L)\left\{ \pi ^{\prime \prime }(\lambda _L)\frac{\partial }{\partial \pi }\tilde{P}_{n,i}(\lambda _L,\pi (\lambda _L))+[\pi ^{\prime }(\lambda _L)]^2\frac{\partial ^2}{\partial \pi ^2}\tilde{P}_{n,i}(\lambda _L,\pi (\lambda _L)) \right\} \right] \\&\qquad +\sum _{n=1}^{\infty }{\tilde{\alpha }}_n\sum _{i=1}^n\frac{i}{n}\left[ \mu _i^{\prime \prime }(\lambda _L)\tilde{P}_{n,i}(\lambda _L,\pi (\lambda _L))\right] =0, \end{aligned} \end{aligned}$$

from which substituting $\lambda _L=0$ and applying Lemma B.1 as well as the definition of $B_n$ gives

B.17 $$\begin{aligned} -\pi ^{\prime \prime }(0)= & {} \sum _{n=1}^{\infty }{\tilde{\alpha }}_n\left[ A_n+B_n-4(n-1)(n-2)\pi (0)^2(1-\pi (0)) \right] \nonumber \\{} & {} + \sum _{n=1}^{\infty }{\tilde{\alpha }}_n\left[ 4(n-1)\pi (0)\pi ^{\prime }(0)+2(n-1)\pi ^{\prime }(0)(2\pi (0)-1) \right] .\qquad \qquad \end{aligned}$$

Differentiating (B.16), we find

$$\begin{aligned} \begin{aligned}&z^{\prime \prime }(\lambda _L)=-\sum _{n=1}^{\infty }{\tilde{\alpha }}_n\frac{1}{n} \left\{ \frac{\partial ^2}{\partial \lambda _L^2}{\hat{\mu }}_{n,1}(\lambda _L,\pi (\lambda _L)) +2\pi ^{\prime }(\lambda _L)\frac{\partial ^2}{\partial \lambda _L\partial \pi }{\hat{\mu }}_{n,1}(\lambda _L,\pi (\lambda _L))\right\} \\&\qquad - \sum _{n=1}^{\infty }{\tilde{\alpha }}_n\frac{1}{n}\left\{ [\pi ^{\prime }(\lambda _L)]^2\frac{\partial ^2}{\partial \pi ^2}{\hat{\mu }}_{n,1}(\lambda _L,\pi (\lambda _L)) +\pi ^{\prime \prime }(\lambda _L)\frac{\partial }{\partial \pi }{\hat{\mu }}_{n,1}(\lambda _L,\pi (\lambda _L))\right\} . \end{aligned} \end{aligned}$$

We hence observe that

B.18 $$\begin{aligned} z^{\prime \prime }(0)=\sum _{n=1}^{\infty }{\tilde{\alpha }}_n\left[ 2(n-1)\pi ^{\prime }(0)(1-2\pi (0))-B_n-\pi ^{\prime \prime }(0)\right] .\nonumber \\ \end{aligned}$$

Combining (B.17) and (B.18) establishes that

B.19 $$\begin{aligned} z^{\prime \prime }(0) =\sum _{n=1}^{\infty }{\tilde{\alpha }}_n\left[ A_n+4(n-1)\pi (0)\pi ^{\prime }(0)-4(n-1)(n-2)\pi (0)^2(1-\pi (0))\right] .\nonumber \\ \end{aligned}$$

Finally, noting that $c(0)=1-\pi (0)$ and that $c^{\prime }(0)+\pi ^{\prime }(0)=\mu _{{\tilde{H}}-1}\pi (0)(1-\pi (0))$, we subtract (B.12) from (B.19) to reach

$$\begin{aligned}\begin{aligned} z^{\prime \prime }(0)-c^{\prime \prime }(0)&=4\pi (0)^2(1-\pi (0)) \left[ \mu ^2_{{\tilde{H}}-1}-\sum _{n=1}^{\infty }{\tilde{\alpha }}_n(n-1)(n-2)\right] \\&=4\pi (0)^2(1-\pi (0))\left[ (\textrm{E}[{\tilde{H}}-1])^2-\textrm{E}[({\tilde{H}}-1)({\tilde{H}}-2)]\right] \\&=4\pi (0)^2(1-\pi (0))\left[ \textrm{E}[{\tilde{H}}-1]-\textrm{var}({\tilde{H}})\right] . \end{aligned} \end{aligned}$$

This establishes Theorem 4.8. $\square$

### B.3 Proof of Theorem 4.11 ($n=3$)

We begin by making the notation in Theorem 4.11 explicit for the case $n=3$. For $i\in \{0,1,2,3\}$, $P_i^{D}$ is the probability of *i* members being infected in a household of size 3 during an epidemic in the households model in which a proportion *z* are infected in the first epidemic. Similarly, $P_i^U$ is the probability of a household containing *i* vaccinated individuals, assuming vaccination uniformly at random with probability *z*. Note that $P_i^{D}, P_i^U$ and *z* are considered as functions of $\pi \in (0,1)$. We begin with a preliminary lemma. Write $q_1=\phi (\lambda _L)$ and $q_2=\phi (2\lambda _L)$ and observe that $0<q_2<q_1<1$ for all $\lambda _L>0$. Further, $P_0^U=(1-z)^3$, $P_1^U=3z(1-z)^2$ and $P_2^U=3z^2(1-z)$. The system in (2.5), or direct calculation, gives $P_0^D=\pi ^3$, $P_1^D=3\pi ^2(1-\pi )q_2$ and $P_2^D=3\pi (1-\pi )q_1\left( 2\pi (q_1-q_2)+(1-\pi )q_1\right)$.

#### Lemma B.2

Let $A=3(P_0^D-P_0^U)$, $B=2(P_1^D-P_1^U)$ and $C=P_2^D-P_2^U$. Then $A-C>0$.

#### Proof

Considering the remaining susceptibles, note that

B.20 $$\begin{aligned} 3P_0^D+2P_1^D+P_2^D=3(1-z)\qquad \text {and}\qquad 3P_0^U+2P_1^U+P_2^U=3(1-z).\nonumber \\ \end{aligned}$$

Hence, $$A+B+C=0$$, so

$$\begin{aligned} A-C=2A+B=6\left( \pi ^3+\pi ^2(1-\pi )q_2-(1-z)^2 \right) . \end{aligned}$$

Using the first equation in (B.20),

$$\begin{aligned} 1-z=\pi \left( \pi ^2+2\pi (1-\pi )[q_2(1-q_1)+q_1^2]+(1-\pi )^2q_1^2\right) \equiv \pi h(\pi ), \end{aligned}$$

so $A>C$ if and only if $f(\pi )>0$, where

$$\begin{aligned} f(\pi )=\pi +(1-\pi )q_2-h(\pi )^2. \end{aligned}$$

Jensen’s inequality gives $q_2>q_1^2$, so $f(0)=q_2-q_1^4>0$. Further, $f(1)=0$, so $f(\pi )>0$ for $\pi \in (0,1)$ if *f* is concave on [0, 1]. Now,

$$\begin{aligned} f^{\prime \prime }(\pi )=-2[h(\pi )h^{\prime \prime }(\pi )+h^{\prime }(\pi )^2], \end{aligned}$$

whilst we also have $h(\pi )>0$ and

$$\begin{aligned} h^{\prime \prime }(\pi )=2(1-q_1)(1+q_1-2q_2)>0. \end{aligned}$$

Thus $f^{\prime \prime }(\pi )<0$ for $\pi \in [0,1]$, so *f* is concave on [0, 1] and $A>C$, as required. $\square$

We now prove Theorem 4.11 in the case $n=3$.

#### Proof

We show that ${\hat{R}}_{DI} (z) > {\hat{R}}_U (z)$, from which the desired result follows.

We have that

B.21 $$\begin{aligned} \frac{3}{\lambda _G\textrm{E}[T_I]} \left( {\hat{R}}_{DI}(z)-{\hat{R}}_U(z) \right) =&A\mu _3(\lambda _L) +B\mu _2(\lambda _L)+C\mu _1(\lambda _L) \nonumber \\ =&A[\mu _3(\lambda _L)-\mu _2(\lambda _L)]-C[\mu _2(\lambda _L)-\mu _1(\lambda _L)]. \end{aligned}$$

Using (2.2) gives $\mu _1(\lambda _L)=1$, $\mu _2(\lambda _L)=2-q_1$, and $\mu _3(\lambda _L)=3-2q_1(q_1-q_2)-2q_2$. Therefore

B.22 $$\begin{aligned} \mu _3(\lambda _L)-\mu _2(\lambda _L)-[\mu _2(\lambda _L)-\mu _1(\lambda _L)]=2(q_1-q_2)(1-q_1)>0. \end{aligned}$$

Since (by Lemma B.2) $A>C$, it follows from (B.21) and (B.22) that ${\hat{R}}_{DI}(z)>{\hat{R}}_U(z)$. $\square$

## C Early exponential growth rate *r* of SEIR households model

The early exponential growth rate (Malthusian parameter) *r* is defined as follows. Consider the branching process of infected households described in Sect. 2.2. Then the Malthusian parameter, *r*, for the branching process satisfies

C.1 $$\begin{aligned} \int _{0}^{\infty }\textrm{e}^{-rt}\beta (t)\,\textrm{d}t=1, \end{aligned}$$

where $\beta (t)$ is the mean rate of global contacts emanating from a typical single-household epidemic, *t* time units after the household is infected. For many choices of distributions for $T_E$ and $T_I$, the left-hand-side of (C.1) is not tractable; in Ball et al. (2016) Section 2.8, ways to approximate it are considered. We restrict attention to the case $T_E \sim \textrm{Exp}(\delta )$ and $T_I \sim \textrm{Exp}(\gamma )$, in which case the dynamics are Markovian and the left-hand side of (C.1) can be computed as outlined below, cf. Pellis et al. (2011), Section 4.2, which considers an SIR model.

In the branching process in Sect. 2.2, individuals correspond to infected households, and an individual gives birth whenever a global contact arises from the corresponding single-household epidemic. We extend Ball and Shaw (2015), Section 4.1, to include an exposed state. For $n=1,2,\dots$, let ${\tilde{E}}^{(n)}_H$ denote a typical single-household epidemic in a household of size *n*, initiated by one member becoming exposed at time $t=0$. For $t\ge 0$, let $S_H^{(n)}(t)$, $E_H^{(n)}(t)$ and $I_H^{(n)}(t)$ denote respectively the number of susceptibles, exposed and infected individuals in ${\tilde{E}}_H^{(n)}$ at time *t*. Let ${\mathscr {F}}^{(n)}=\{ (s,e,i) \in {\mathbb {Z}}_+^3: s+e+i \le n\}$ be the set of possible household states for a household of size *n*. For $(s,e,i) \in {\mathscr {F}}^{(n)}$ and $t \ge 0$, let $p^{(n)}_{s,e,i}(t)=\textrm{P}\left( (S_H^{(n)}(t),E_H^{(n)}(t),I_H^{(n)}(t))=(s,e,i)\right)$. Let $S_n=\left|{\mathscr {F}}^{(n)}\right|=\frac{n}{6}\left( n^2+6n+11\right)$. For a given household of size *n*, we have, in an obvious notation,

$$\begin{aligned} \beta ^{(n)}(t)=\lambda _G\sum _{(s,e,i)\in {\mathscr {F}}^{(n)}}ip^{(n)}_{s,e,i}(t), \end{aligned}$$

which after conditioning on the size of a typical household in the approximating branching process yields

$$\begin{aligned} \beta (t)=\lambda _G\sum _{n=1}^{\infty }{\tilde{\alpha }}_n\sum _{(s,e,i)\in {\mathscr {F}}^{(n)}}ip^{(n)}_{s,e,i}(t). \end{aligned}$$

Hence, using (C.1), *r* satisfies

C.2 $$\begin{aligned} \lambda _G\sum _{n=1}^{\infty }{\tilde{\alpha }}_n\sum _{(s,e,i)\in {\mathscr {F}}^{(n)}}i{\tilde{p}}^{(n)}_{s,e,i}(r)=1, \end{aligned}$$

where

$$\begin{aligned} {\tilde{p}}^{(n)}_{s,e,i}(r)=\int _0^\infty \textrm{e}^{-rt}p^{(n)}_{s,e,i}(t)\,\textrm{d}t. \end{aligned}$$

To calculate ${\tilde{p}}^{(n)}_{s,e,i}(r)$, let $Q^{(n)}=\left[ q^{(n)}_{i,j}\right]$ be the $S_n \times S_n$ transition-rate matrix of ${\tilde{E}}_H^{(n)}$, with states labelled such that $(n-1,1,0)$ is state 1. Suppose $k\in \{1,2,\dots ,S_n\}$ corresponds to the state $(s,e,i)\in {\mathscr {F}}^{(n)}$. Then

$$\begin{aligned} p^{(n)}_{s,e,i}(t)=\left( \textrm{e}^{tQ^{(n)}} \right) _{1,k}, \end{aligned}$$

where $\textrm{e}^A=\sum _{i=0}^\infty \frac{A^i}{i!}$ denotes the matrix exponential. Hence,

C.3 $$\begin{aligned} {\tilde{p}}^{(n)}_{s,e,i}(r)=\left( [rI_{S_n}-Q^{(n)}]^{-1} \right) _{1,k} \end{aligned}$$

and *r* can then be computed using (C.2) and (C.3).

Further, if $\delta , \gamma , \lambda _L$ and the household structure are known and fixed then, for a given value of the early growth rate *r*, the corresponding $\lambda _G$ is readily obtained using  (C.2) and (C.3).

## D Household data

The household data used for Finland, Sweden, Italy and the UK are taken from Eurostat (EU-SILC survey 2022). The remaining data are from the United Nations household size and composition around the world data booklet (2017) and for these countries the proportion of households of each type is not readily available; the information available is $\textrm{E}[H]$ as well as $P(H=1)$, $P(H=2 \text { or } H=3)$, $\textrm{P}(H=4 \text { or } H=5)$ and $\textrm{P}(H>5)$. For these countries we then wish to estimate, for $x=1,2,\dots$, $\textrm{P}(H=x)$, from which we can also estimate $\textrm{P}({\tilde{H}}=x)={\tilde{\alpha }}_x$. To do so, we use maximum likelihood estimation assuming *H* has a shifted negative binomial distribution having probability mass function

$$\begin{aligned} f_{r,p}(x)=\frac{\Gamma (x)}{\Gamma (r)\Gamma (x-r+1)} p^r (1-p)^{x-1}, \qquad x=1,2,\dots , \end{aligned}$$

where $r \in (0,\infty )$ and $p\in (0,1)$ are parameters to be estimated. Since $\textrm{E}[H]$ is known, this reduces to a one-parameter maximisation (for *r*, say) using $\textrm{E}[H]=1+\frac{r(1-p)}{p}$. Then, letting ${\tilde{p}}={\tilde{p}}(r) =\frac{r}{\textrm{E}[H]+r-1}$ and $x_i$ be the proportion of households of size *i* ($i=1,2,\dots$), we have the likelihood

$$\begin{aligned} \begin{aligned} L(r)=&\left[ f_{r,{\tilde{p}}}(1)\right] ^{x_1}\left[ f_{r,{\tilde{p}}}(2)+f_{r,{\tilde{p}}}(3)\right] ^{x_2+x_3}\left[ f_{r,{\tilde{p}}}(4)+f_{r,{\tilde{p}}}(5)\right] ^{x_4+x_5} \\&\qquad \times \left[ 1-\sum _{i=1}^5f_{r,{\tilde{p}}}(i)\right] ^{1-\sum _{i=1}^5 x_i}, \end{aligned} \end{aligned}$$

which can be optimised numerically over *r* to find the maximum likelihood estimate of *r*.

We note also that calculating any herd immunity level when the household size distribution has unbounded support requires truncation of that distribution. The truncation point must be chosen carefully to ensure that results are insensitive to the precise choice. For the household size distributions that we use we find that the approximation ${\tilde{\alpha }}_{15}=1-\sum _{n=1}^{14}{\tilde{\alpha }}_n$ is sufficient.
